# Supplementary material for: Haplotype-based analysis distinguishes maternal-fetal genetic contribution to pregnancy-related outcomes
Source: PLoS Genet. 2025 Mar 10;21(3):e1011575. doi: 10.1371/journal.pgen.1011575 (PMC11918446; doi:10.1371/journal.pgen.1011575)
Supplement: S1 Text — (DOCX) [file pgen.1011575.s001.docx]

# **Supporting Data**

***Title:* Haplotype-based analysis distinguishes maternal-fetal genetic contribution to pregnancy-related outcomes**

***Short Title:* Resolving maternal-fetal genetic contribution to pregnancy-related outcomes**

Amit K. Srivastava^1^, Julius Juodakis^3^, Pol Sole-Navais^3^, Jing Chen^4^, Jonas Bacelis^3,5^, Kari Teramo^6†^, Mikko Hallman^7^, Pal R. Njølstad^8,9,10^, David M. Evans^11,12^, Bo Jacobsson^13,14^, Louis J. Muglia^1,2^, Ge Zhang^1,2*^

**Affiliations**

1. Division of Human Genetics, Center for Prevention of Preterm Birth, Perinatal Institute and March of Dimes Prematurity Research Center Ohio Collaborative, Cincinnati Children’s Hospital Medical Center, Cincinnati, Ohio, United States of America
2. Department of Pediatrics, University of Cincinnati College of Medicine, Cincinnati, Ohio, United States of America
3. Department of Obstetrics and Gynecology, Institute of Clinical Sciences, Sahlgrenska Academy, University of Gothenburg, Gothenburg, Sweden
4. Division of Biomedical Informatics, Cincinnati Children’s Hospital Medical Center, Cincinnati, Ohio, United States of America
5. Region Västra Götaland, Sahlgrenska University Hospital, Department of Obstetrics and Gynecology, Gothenburg, Sweden
6. Obstetrics and Gynecology, University of Helsinki and Helsinki University Hospital, Helsinki, Finland
7. PEDEGO Research Unit and Medical Research Center Oulu, University of Oulu and Department of Children and Adolescents, Oulu University Hospital, Oulu, Finland
8. KG Jebsen Center for Diabetes Research, Department of Clinical Science, University of Bergen, Bergen, Norway
9. Division of Health Data and Digitalization, Department of Genetics and Bioinformatics, Norwegian Institute of Public Health, Oslo, Norway
10. Center for Medical Genetics and Molecular Medicine, Haukeland University Hospital, Bergen, Norway
11. Institute for Molecular Bioscience, Frazer Institute, The University of Queensland, Brisbane, Australia
12. Medical Research Council Integrative Epidemiology Unit, University of Bristol, United Kingdom
13. Department of Obstetrics and Gynecology, Sahlgrenska Academy, University of Gothenburg, Gothenburg, Sweden
14. Department of Genetics and Bioinformatics, Area of Health Data and Digitalization, Norwegian Institute of Public Health, Oslo, Norway

*****[**Ge.Zhang@cchmc.org**](mailto:Ge.Zhang@cchmc.org)

† Deceased

**Contents**

[Supporting Data 1](#_Toc189652723)

[I) Description of datasets1](#_Toc189652724)

[**A)** **Avon Longitudinal Study of Parents and Children (ALSPAC)** 1](#_Toc189652725)

[**B) Hyperglycemia and Adverse Pregnancy Outcome (HAPO)** 1](#_Toc189652726)

[**C) Finnish Dataset (FIN)** 2](#_Toc189652727)

[**D) Danish National Birth Cohort (DNBC)** 2](#_Toc189652728)

[**E) Norwegian Mother and Child Cohort Study (MoBa)** 3](#_Toc189652729)

[II) Quality control of genotypes 4](#_Toc189652730)

[III) Quality control of phenotypes 6](#_Toc189652731)

[IV) Heritability estimation using SNPs with MAF > 0.001, MAF > 0.01 and MAF > 0.05 6](#_Toc189652732)

[V) Replication of heritability estimation 7](#_Toc189652733)

[References 8](#_Toc189652734)

# **Supporting Data**

## **Description of datasets**

### **Avon Longitudinal Study of Parents and Children (ALSPAC)**

ALSPAC is an observational study to investigate the genetic and environmental influence on health and development of parents and their offspring^1,2^. The study was approved by ALSPAC Ethics and law Committee and the local Research Ethics Committees. The study consists of 14,541 pregnancies recruited from Avon County, Bristol, England with expected dates of delivery between April 01’ 1991 and December 31’ 1992. Of these, initial pregnancies, there was a total of 14,676 fetuses, resulting into 14,062 live births and 13,988 children who lived more than 1 year. The women, their partners and children were followed up for 19-22 years with 20 completed questionnaires. The study website contains details of all the data that is available through a fully searchable data dictionary and variable search tool (<http://www.bristol.ac.uk/alspac/researchers/our-data/>). Genotype data of the mothers and children were generated using the Illumina HumanHap550 quad (children) and Illumina human660W quad (mothers). Genotype data consisted of 17,842 participants (either mothers or children), containing 6,305 mother-child pairs, each with 465,740 SNPs genotyped. A total of 5,369 mother-child pairs who passed genotype QC and inclusion/exclusion criteria were included in the analysis (S1 Table; S2A Fig).

### **B) Hyperglycemia and Adverse Pregnancy Outcome (HAPO)**

HAPO is an international study conducted at multiple centers for fetal growth and maternal glucose levels during pregnancy^3^. High quality phenotypic data has been collected from 25,000 pregnant women using standardized protocols, uniform across centers. These women belonged to different racial and socio-demographic backgrounds. We downloaded data of 1,500 mother-child pairs (2,866 samples with genotype data) of European ancestry (phs000096.v2.p1) from dbGaP (<http://www.ncbi.nlm.nih.gov/sites/entrez?db=gap>/) for the current study. Gestational duration in this dataset was determined by last menstrual period (LMP) or ultrasound estimation between 6-24 weeks. After quality control of genotypes and phenotypes, we included 1089 mother-child pairs for further analysis (S1 Table; S2B Fig).

### **C) Finnish Dataset (FIN)**

FIN is a case-control study of spontaneous preterm birth. More than 1,600 mother/child pairs from the Helsinki University Hospitals were recruited between 2004 and 2014^4,5^. The study was approved by the Ethics Committee of Oulu University Hospital and that of Helsinki University Central Hospital. Whole blood samples were collected from all of them after written informed consent and 2,962 blood samples from mothers and children were genotyped. All the studied samples were of Finnish descent. Gestational duration was determined by crown-rump length (CRL) at the first ultrasound screening between 10 and 13 weeks. 1,169 mother-child pairs were selected for further analysis after genotype quality control (QC) procedure and applying the phenotype-based inclusion/exclusion criteria (S1 Table; S2C Fig).

### **D) Danish National Birth Cohort (DNBC)**

DNBC is an epidemiological birth cohort for the study of health outcomes in mothers and their offspring^6^. The study followed more than 100,000 pregnancies between 1996 and 2003, starting in the first trimester of pregnancy. The study was approved by the Danish Scientific Ethical Committee and the Danish Data Protection Agency. We used genotype data (phs000103.v1.p1) generated in two genome-wide association studies - the study of preterm delivery and the study of obesity, available at dbGaP website (<http://www.ncbi.nlm.nih.gov/sites/entrez?db=gap>/). In total, 5,921 mothers and 2,130 infants (1739 mother-child pairs) after QC were utilized for the analysis (S1 Table; S2D Fig). Gestational duration in this dataset was determined by a consensus algorithm combining all available information from multiple sources: self-reported date of last menstrual period (LMP), self-reported delivery date, and gestational duration at birth registered in the Medical Birth Register and the National Patient Register.

### **E) Norwegian Mother and Child Cohort Study (MoBa)**

MoBa is a Norwegian pregnancy study for causes of disease in mothers and their offspring, initiated and followed by the Norwegian Institute of Public Health (NIPH)^7^. The study includes more than 114,000 children, 95,000 mothers and 75,000 fathers recruited between 1999 through 2008 starting from week 17 in pregnancy. The study was approved by The Regional Committee for Medical and Health Research Ethics in South-Eastern, Norway (2009/1387 and 2010/2683 S-6075). An informed written consent was obtained prior to sample collection. For most pregnancies, gestational duration was estimated by anatomical ultrasound at gestational weeks 17–19. However, ultrasound dating could not be obtained for some pregnancies and gestational duration was estimated using LMP. For the current study, we used the mother-child pairs, selected from Version 4 of the MoBa dataset, which included a total of 71,669 pregnancies. We selected singleton live-births from mothers in the age group 20–34 years. Pregnancies involving pre-existing medical conditions, complications during pregnancies and conceived by in-vitro fertilization were excluded from the study. Random sampling was done from two gestational duration ranges 154-258 days (cases) and 273–286 days (controls). In total, 3,121 mothers and children were genotyped. 1,834 mothers and 1,143 children (1080 mother-child pairs) were included in the analysis following genotype QC and phenotype inclusion/exclusion (S1 Table; S2E Fig).

## **Quality control of genotypes**

Genotyping was performed on various Affymetrix and Illumina SNP array platforms. Specifically, genotyping in ALSPAC dataset was conducted on Illumina HumanHap550 quad (children) and Illumina human660W quad (mothers); SNPs in MoBa dataset were genotyped using Illumina Human660W-Quad bead arrays; and SNPs genotyping of the Finnish cohort was done using Affymetrix SNP array 6.0 and different Illumina arrays. HAPO and DNBC datasets were downloaded from dbGaP. HAPO dataset was genotyped on Human610-Quad array; and DNBC samples were genotyped using Illumina Human660W-Quad bead arrays. Genotype calls from Affymetrix SNP array 6.0 were determined using CRLMM algorithm among chips that passed the vendor-suggested QC (contrast QC > 0.4). Similar procedure was used for Illumina SNP arrays in all datasets except FIN where genotype calling was conducted using Illumina’s genotyping module v1.94 in the GenomeStudio v2011.1.

Following genotype calls, we performed genotype QC across all the datasets separately. Genotype QC was performed at individual and marker level using Plink 1.9^8^. Individual-level QC was based on call rate per individual - samples with < 95% SNPs called were excluded from the study, average heterozygosity across all genotypes - samples with substantially high or low heterozygosity were excluded from the study, sex assignment using heterozygosity in X-chromosome SNPs and IBD analysis. Homogeneity of samples and genetic ancestry was determined by principal components analysis (PCA) anchored by 1000 Genomes reference samples. Individuals with non-European ancestry were excluded. Marker level QC was performed on the basis of call rate - SNPs with call rate <98% were excluded from the study, minor allele frequency (MAF) – SNPs with MAF < 0.01 were excluded from the study and Hardy-Weinberg Equilibrium (HWE) – SNPs showing significant deviation from HWE (P < 5×10^-6^) were excluded^9^.

The genotype data of the mothers and children who passed QC was phased together using Shapeit2 to infer maternal transmitted alleles (m1), maternal non-transmitted alleles (m2) and paternal transmitted alleles (p1)^10^. While phasing mother-child together, SHAPEIT automatically detects their relationship provided in the input file. The first allele in child is maternal transmitted whereas second allele is paternal transmitted. In the case of trios, the first allele in child is paternal transmitted whereas second allele is maternal transmitted. Then, we imputed the pre-phased mother-child data for missing SNPs using haplotype reference consortium (HRC) data containing 64,976 haplotypes with 39,235,157 SNPs^11^. Phased genotype data was uploaded to Sanger Imputation Server where imputation was done using Positional Burrow-Wheeler Transformation (PBWT)^12^. Since genotype data of the FIN dataset was generated using multiple platforms, the pre-phasing and imputation were done separately for each platform. Later, all samples with imputed genotypes were merged and re-phasing of SNPs with MAF > 0.05 was done using Shapeit2. Pre-phased Finnish dataset was imputed in the same way as earlier. From imputed genotypes, three different sets of SNPs were selected based on MAF cutoff (all polymorphic SNPs, SNPs with MAF > 0.001 and SNPs with MAF > 0.01) in individual datasets separately. Mothers were considered as founders and the same set of SNPs were selected from children in each dataset. Then, a common set of SNPs were selected across datasets in each MAF cutoff category. Eventually, genotype data of common sets of SNPs from each dataset were pooled together for further analysis (S2, S3 Tables).

## **III) Quality control of phenotypes**

In the current study, we focused on pregnancy outcomes including gestational duration and fetal size measurements at birth (birth weight, birth length and head circumference). All traits in the current study were available only in two datasets – ALSPAC and HAPO. Birth weight was accessible in all datasets except MoBa. Birth length was accessible in ALSPAC, HAPO and FIN whereas head circumference was available in ALSPAC and HAPO. We considered gestational duration as primary trait while doing quality control (QC) of phenotypes. We only included spontaneous, singleton pregnancies whose children were alive > 1 year and both parents self-reported European ancestry. We excluded pregnancies with history of medical conditions influencing pre-term birth such as pre-pregnancy diabetes, hypertension, placental and congenital anomalies. We also excluded pregnancies with any risk factors for pre-term birth during pregnancies such as gestational diabetes, gestational hypertension and preeclampsia (S4 Table).

## **IV) Heritability estimation using SNPs with MAF > 0.001, MAF > 0.01 and MAF > 0.05**

Besides all polymorphic SNPs, $\hat{h}^{2}$ was also estimated using SNPs with MAF > 0.001, MAF > 0.01 and MAF > 0.05 in pooled dataset of 10,375 unrelated mother-child pairs (relatedness coefficient cutoff > 0.05). The focus of these analyses was to estimate the contribution of very rare, rare, common and very common variants. We estimated $\hat{h}^{2}$ of gestational duration and gestational duration adjusted birth weight, birth length and head circumference using conventional GCTA^13,14^, contemporary M-GCTA^15,16^ and newly developed H-GCTA approach by utilizing REML^17,18^ implemented through GCTA and LDAK. We used different models accounting for the influence of pair-wise linkage disequilibrium (LD) and minor allele frequency (MAF) on $\hat{h}^{2}$ estimates (S24-26 Tables). Results obtained from these analyses were like the results obtained through analysis using all polymorphic SNPs. These results indicated that exclusion of very rare and rare variants does not substantially change heritability estimates.

## **V) Replication of heritability estimation**

We also replicated analysis using our approach through GREML (α = -1.0) implemented through GCTA in another Nordic cohort – HARVEST from Norway. In this study, approximately 8,000 mother-child pairs were available after quality control. Standard procedures similar to MoBa were used while blood sample collection and genotyping. Only gestational duration was available from the dataset. We used common SNPs with MAF > 0.01 for SNP-based narrow-sense heritability ($\hat{h}^{2}$) estimation. Similar, to discovery cohorts, gestational duration was adjusted for fetal sex. We observed genetic variance attributable to maternal transmitted alleles (m1) – 7.7% (S.E.: 3.8%, p value = 2.22E-02), maternal non-transmitted alleles (m2) – 4.3% (S.E.: 3.8%, p value = 1.30E-01) and paternal transmitted alleles (P1) – 2.5% (S.E.: 3.7%, p value = 2.49E-02). Results from HARVEST dataset confirmed our findings that genetic variance of gestation duration is mainly contributed by maternal genome (S7 Fig; S27 Table).

## **References**

1. Fraser, A. *et al.* Cohort Profile: the Avon Longitudinal Study of Parents and Children: ALSPAC mothers cohort. *Int J Epidemiol* **42**, 97-110 (2013).

2. Boyd, A. *et al.* Cohort Profile: the 'children of the 90s'--the index offspring of the Avon Longitudinal Study of Parents and Children. *Int J Epidemiol* **42**, 111-27 (2013).

3. Group, H.S.C.R. The Hyperglycemia and Adverse Pregnancy Outcome (HAPO) Study. *Int J Gynaecol Obstet* **78**, 69-77 (2002).

4. Zhang, G. *et al.* Genetic Associations with Gestational Duration and Spontaneous Preterm Birth. *N Engl J Med* **377**, 1156-1167 (2017).

5. Plunkett, J. *et al.* An evolutionary genomic approach to identify genes involved in human birth timing. *PLoS Genet* **7**, e1001365 (2011).

6. Olsen, J. *et al.* The Danish National Birth Cohort--its background, structure and aim. *Scand J Public Health* **29**, 300-7 (2001).

7. Magnus, P. *et al.* Cohort Profile Update: The Norwegian Mother and Child Cohort Study (MoBa). *Int J Epidemiol* **45**, 382-8 (2016).

8. Chang, C.C. *et al.* Second-generation PLINK: rising to the challenge of larger and richer datasets. *Gigascience* **4**, 7 (2015).

9. Winkler, T.W. *et al.* Quality control and conduct of genome-wide association meta-analyses. *Nat Protoc* **9**, 1192-212 (2014).

10. Delaneau, O., Marchini, J. & Zagury, J.F. A linear complexity phasing method for thousands of genomes. *Nat Methods* **9**, 179-81 (2011).

11. McCarthy, S. *et al.* A reference panel of 64,976 haplotypes for genotype imputation. *Nat Genet* **48**, 1279-83 (2016).

12. Durbin, R. Efficient haplotype matching and storage using the positional Burrows-Wheeler transform (PBWT). *Bioinformatics* **30**, 1266-72 (2014).

13. Yang, J., Lee, S.H., Goddard, M.E. & Visscher, P.M. GCTA: a tool for genome-wide complex trait analysis. *Am J Hum Genet* **88**, 76-82 (2011).

14. Yang, J. *et al.* Common SNPs explain a large proportion of the heritability for human height. *Nat Genet* **42**, 565-9 (2010).

15. Eaves, L.J., Pourcain, B.S., Smith, G.D., York, T.P. & Evans, D.M. Resolving the effects of maternal and offspring genotype on dyadic outcomes in genome wide complex trait analysis ("M-GCTA"). *Behav Genet* **44**, 445-55 (2014).

16. Qiao, Z. *et al.* Introducing M-GCTA a Software Package to Estimate Maternal (or Paternal) Genetic Effects on Offspring Phenotypes. *Behav Genet* (2019).

17. Lee, S.H. & van der Werf, J.H. An efficient variance component approach implementing an average information REML suitable for combined LD and linkage mapping with a general complex pedigree. *Genet Sel Evol* **38**, 25-43 (2006).

18. Thompson, H.D.P.a.R. Recovery of Inter-Block Information when Block Sizes are Unequal. *Biometrika* **58**, 545-554 (1971).
