# Supplementary material for: Haplotype-based analysis distinguishes maternal-fetal genetic contribution to pregnancy-related outcomes
Source: PLoS Genet. 2025 Mar 10;21(3):e1011575. doi: 10.1371/journal.pgen.1011575 (PMC11918446; doi:10.1371/journal.pgen.1011575)
Supplement: S1 Table — Number of pregnancies and genotypes present in individual datasets. a) Number of genotypes typed in each dataset b) number of genotypes passed through genotype QC; c) number of pregnancies after genotype QC and phenotype inclusion/exclusion. (DOCX) [file pgen.1011575.s002.docx]

# **S1 Table: Genotype and Phenotype records in datasets**

| **Record** |  | **Datasets** | | | | | **Total** |
| --- | --- | --- | --- | --- | --- | --- | --- |
|  |  | **ALSPAC** | **HAPO** | **FIN** | **DNBC** | **MoBa** |  |
| **Phenotype** | **Count** | 15443 | 1507 | 1644 | 2068 | 2035 | 22697 |
| **Genotype** | **Typed** | 17842 | 2866 | 2962 | 3886 | 3120 | 30676 |
|  | **Passed** | 17435 | 2743 | 2710 | 3799 | 2979 | 29666 |
| **Filtered** | **Count** | 9806 | 1266 | 1371 | 2038 | 1933 | 16414 |
|  | **Mother** | 7603 | 1203 | 1322 | 1912 | 1804 | 13844 |
|  | **Fetus** | 7587 | 1152 | 1217 | 1865 | 1134 | 12955 |
|  | **Duo** | 5369 | 1089 | 1169 | 1739 | 1009 | **10375** |
