# Supplementary material for: Haplotype-based analysis distinguishes maternal-fetal genetic contribution to pregnancy-related outcomes
Source: PLoS Genet. 2025 Mar 10;21(3):e1011575. doi: 10.1371/journal.pgen.1011575 (PMC11918446; doi:10.1371/journal.pgen.1011575)
Supplement: S2 Table — Number of imputed sites using Haplotype Reference Consortium (HRC), polymorphic SNPs in individual datasets and common set of SNPs across all available datasets. In individual datasets, mothers were considered as founders for each MAF cutoff category and corresponding children were selected. Final analysis was performed using pooled data and common set of SNPs across all datasets. (DOCX) [file pgen.1011575.s003.docx]

# **S2 Table: Genotype information in individual datasets**

| **Record** | **Datasets** | | | | | **Common Set** |
| --- | --- | --- | --- | --- | --- | --- |
|  | **ALSPAC** | **HAPO** | **FIN** | **DNBC** | **MoBa** |  |
| Imputed SNPs | 39131578 | | | | | |
| All Polymorphic SNPs | 22660040 | 17506086 | 14123671 | 16660771 | 15015991 | 11017281 |
| SNPs with MAF > 0.001 | 11614189 | 11790056 | 11174353 | 11470397 | 11290187 | 9273676 |
| SNPs with MAF > 0.01 | 7626580 | 7657614 | 7842149 | 7638261 | 7636920 | 6926557 |
