# Supplementary material for: Haplotype-based analysis distinguishes maternal-fetal genetic contribution to pregnancy-related outcomes
Source: PLoS Genet. 2025 Mar 10;21(3):e1011575. doi: 10.1371/journal.pgen.1011575 (PMC11918446; doi:10.1371/journal.pgen.1011575)
Supplement: S3 Table — Number of samples with gestational duration, birth weight, birth length and head circumference in the pooled data; a) without relatedness coefficient cut-off, b) with relatedness coefficient cut-off < 0.05. For mother-child pairs with relatedness coefficient cutoff < 0.05, common set of mother-child pairs were selected from GRMs based on mother’s genotypes, children’s genotypes, maternal transmitted alleles (m1), maternal non-transmitted alleles (m2) and paternal transmitted alleles (p1). (DOCX) [file pgen.1011575.s004.docx]

# **S3 Table: Phenotype information in pooled dataset**

| **Trait** | **Pooled Data** | | | | |
| --- | --- | --- | --- | --- | --- |
|  |  | **All Polymorphic SNPs** | **SNPs with MAF > 0.1%** | **SNPs with MAF > 1%** | **SNPs with MAF > 5%** |
| **Gestational Duration (days)** | No relatedness cut-off^a^ | 10375 | | | |
|  | relatedness < 0.05^b^ | 9437 | 9475 | 9457 | 9408 |
| **Birth Weight (gm)** | No relatedness cut-off^a^ | 9287 | | | |
|  | relatedness < 0.05^b^ | 8383 | 8414 | 8394 | 8345 |
| **Birth Length (cm)** | No relatedness cut-off^a^ | 6571 | | | |
|  | relatedness < 0.05^b^ | 5829 | 5861 | 5841 | 5794 |
| **Head Circumference (cm)** | No relatedness cut-off^a^ | 5459 | | | |
|  | relatedness < 0.05^b^ | 4815 | 4806 | 4796 | 4790 |
