# Supplementary material for: Haplotype-based analysis distinguishes maternal-fetal genetic contribution to pregnancy-related outcomes
Source: PLoS Genet. 2025 Mar 10;21(3):e1011575. doi: 10.1371/journal.pgen.1011575 (PMC11918446; doi:10.1371/journal.pgen.1011575)
Supplement: S4 Table — Descriptive statistics of gestational duration, birth weight, birth length and head circumference in ALSPAC, HAPO, FIN, DNBC and MoBa. All four traits were available only in two datasets, namely ALSPAC and HAPO. (DOCX) [file pgen.1011575.s005.docx]

# **S4 Table: Phenotype summary in individual datasets**

| **Dataset** | **Descriptive Measure** | **Gestational Duration (days)** | | **Birth Weight (gram)** | | **Birth Length (cm)** | | **Head Circumference (cm)** | |
| --- | --- | --- | --- | --- | --- | --- | --- | --- | --- |
|  |  | **Male** | **Female** | **Male** | **Female** | **Male** | **Female** | **Male** | **Female** |
| ALSPAC | count | 2690 | 2694 | 2659 | 2659 | 2154 | 2172 | 2183 | 2198 |
|  | min | 189 | 189 | 980 | 844 | 36 | 38.9 | 20.7 | 20.3 |
|  | max | 301 | 301 | 5300 | 5040 | 61 | 60 | 54 | 39.5 |
|  | mean | 276.9 | 278.2 | 3520.2 | 3399.2 | 51.2 | 50.4 | 35.2 | 34.5 |
|  | sd | 12.2 | 10.6 | 526.1 | 466.5 | 2.3 | 2.1 | 1.5 | 1.3 |
|  |  |  |  |  |  |  |  |  |  |
| HAPO | count | 550 | 539 | 550 | 539 | 550 | 539 | 550 | 539 |
|  | min | 259 | 259 | 1820 | 2066 | 37.3 | 44.3 | 30.15 | 29 |
|  | max | 296 | 300 | 5100 | 5018 | 58.3 | 58.4 | 54.05 | 38 |
|  | mean | 279.9 | 280.6 | 3519.4 | 3353.1 | 51.0 | 50.2 | 35.2 | 34.6 |
|  | sd | 8.1 | 8.1 | 502.6 | 473.9 | 2.3 | 2.1 | 1.7 | 1.3 |
|  |  |  |  |  |  |  |  |  |  |
| FIN | count | 606 | 564 | 606 | 564 | 605 | 563 |  |  |
|  | min | 173 | 191 | 760 | 915 | 32 | 34 |  |  |
|  | max | 294 | 294 | 5150 | 4720 | 58 | 56 |  |  |
|  | mean | 271.2 | 273.0 | 3340.1 | 3260.0 | 49.5 | 48.9 |  |  |
|  | sd | 20.5 | 19.5 | 707.5 | 630.3 | 3.2 | 2.9 |  |  |
|  |  |  |  |  |  |  |  |  |  |
| DNBC | count | 908 | 831 | 904 | 822 |  |  |  |  |
|  | min | 171 | 168 | 530 | 521 |  |  |  |  |
|  | max | 294 | 298 | 5270 | 5100 |  |  |  |  |
|  | mean | 264.1 | 264.1 | 3228.9 | 3103.6 |  |  |  |  |
|  | sd | 23.3 | 23.4 | 845.0 | 796.8 |  |  |  |  |
|  |  |  |  |  |  |  |  |  |  |
| MoBa^Ϯ^ | count | 503 | 506 |  |  |  |  |  |  |
|  | min | 182 | 172 |  |  |  |  |  |  |
|  | max | 286 | 286 |  |  |  |  |  |  |
|  | mean | 263.6 | 266.1 |  |  |  |  |  |  |
|  | sd | 18.6 | 17.6 |  |  |  |  |  |  |

Ϯ – Only gestational duration data was available to in MoBa dataset.
