# Supplementary material for: Haplotype-based analysis distinguishes maternal-fetal genetic contribution to pregnancy-related outcomes
Source: PLoS Genet. 2025 Mar 10;21(3):e1011575. doi: 10.1371/journal.pgen.1011575 (PMC11918446; doi:10.1371/journal.pgen.1011575)
Supplement: S5 Table — h^2 of simulated maternal traits from ALSPAC dataset, estimated through conventional GCTA, M-GCTA and H-GCTA approach. Each approach was fitted using GREML (α = -0.25, -1.0), LDAK-Thin (α = -0.25, -1.0) and LDAK-Weights (α = -0.25, -1.0). For GCTA, M is the GRM generated from maternal genotypes (m), and F is the GRM generated from fetal genotypes (f). For M-GCTA, M’ represents the genetic relationship matrix of mothers; G represents genetic relationship matrix of children and D represents mother-child covariance matrix. For H-GCTA, M1 is the GRM generated from maternal transmitted alleles (m1), M2 is the GRM generated from maternal non-transmitted alleles (m2), and P1 is the GRM generated from paternal transmitted alleles (p1). A total of 100 replicates of each phenotype were simulated using empirical genotypes of ALSPAC dataset. P-values were calculated using z test statistics (two sided). (DOCX) [file pgen.1011575.s006.docx]

# **S5 Table: SNP-based heritability of simulated maternal traits from ALSPAC dataset**

| **h^2^ of maternal traits** | | | GREML (alpha = -1.0) | | | GREML (alpha = -0.25) | | | | LDAK-Thin (alpha = -1.0) | | | | LDAK-Thin (alpha = -0.25) | | | | LDAK-Weights (alpha = -1.0) | | | | LDAK-Weights (alpha = -0.25) | | | |  |
| --- | --- | --- | --- | --- | --- | --- | --- | --- | --- | --- | --- | --- | --- | --- | --- | --- | --- | --- | --- | --- | --- | --- | --- | --- | --- | --- |
| MAF Cut-off | Approach | GRM | ĥ^2^ | S.E. | p-val | | ĥ^2^ | SD | p-val | | ĥ^2^ | SD | p-val | | ĥ^2^ | SD | p-val | | ĥ^2^ | SD | p-val | | ĥ^2^ | SD | p-val | |
| All Polymorphic SNPs | GCTA | M | 0.4700 | 0.0899 | 1.71E-07 | | 0.2623 | 0.0516 | 3.66E-07 | | 0.6176 | 0.1473 | 2.77E-05 | | 0.3011 | 0.0773 | 9.72E-05 | | 0.3742 | 0.1759 | 3.34E-02 | | 0.4529 | 0.1508 | 2.68E-03 | |
|  |  | F | 0.1097 | 0.0899 | 2.22E-01 | | 0.0726 | 0.0516 | 1.59E-01 | | 0.0581 | 0.1473 | 6.93E-01 | | 0.0526 | 0.0773 | 4.96E-01 | | -0.0751 | 0.1759 | 6.69E-01 | | 0.0599 | 0.1508 | 6.91E-01 | |
|  | M-GCTA | M' | 0.5405 | 0.1121 | 1.41E-06 | | 0.2835 | 0.0663 | 1.92E-05 | | 0.8004 | 0.1864 | 1.76E-05 | | 0.3406 | 0.0999 | 6.50E-04 | | 0.6062 | 0.2305 | 8.53E-03 | | 0.5970 | 0.1948 | 2.17E-03 | |
|  |  | G | 0.0491 | 0.1013 | 6.27E-01 | | 0.0243 | 0.0672 | 7.18E-01 | | 0.0025 | 0.1737 | 9.88E-01 | | -0.0068 | 0.0915 | 9.41E-01 | | -0.1264 | 0.2420 | 6.01E-01 | | 0.0450 | 0.2014 | 8.23E-01 | |
|  |  | D | -0.0842 | 0.0895 | 3.47E-01 | | -0.0271 | 0.0569 | 6.34E-01 | | -0.1841 | 0.1523 | 2.27E-01 | | -0.0366 | 0.0805 | 6.50E-01 | | -0.1877 | 0.2018 | 3.52E-01 | | -0.1473 | 0.1505 | 3.28E-01 | |
|  | H-GCTA | M1 | 0.2416 | 0.0909 | 7.84E-03 | | 0.1363 | 0.0592 | 2.13E-02 | | 0.2304 | 0.1585 | 1.46E-01 | | 0.1193 | 0.0851 | 1.61E-01 | | 0.0352 | 0.1878 | 8.52E-01 | | 0.2157 | 0.1666 | 1.95E-01 | |
|  |  | M2 | 0.2493 | 0.1001 | 1.28E-02 | | 0.1331 | 0.0595 | 2.52E-02 | | 0.3772 | 0.1643 | 2.16E-02 | | 0.1559 | 0.0874 | 7.45E-02 | | 0.3005 | 0.1832 | 1.01E-01 | | 0.3102 | 0.1608 | 5.37E-02 | |
|  |  | P1 | 0.0076 | 0.0756 | 9.20E-01 | | -0.0091 | 0.0473 | 8.48E-01 | | -0.0092 | 0.1279 | 9.43E-01 | | -0.0016 | 0.0666 | 9.81E-01 | | -0.0367 | 0.1619 | 8.20E-01 | | 0.0094 | 0.1368 | 9.46E-01 | |
