# Supplementary material for: Haplotype-based analysis distinguishes maternal-fetal genetic contribution to pregnancy-related outcomes
Source: PLoS Genet. 2025 Mar 10;21(3):e1011575. doi: 10.1371/journal.pgen.1011575 (PMC11918446; doi:10.1371/journal.pgen.1011575)
Supplement: S6 Table — h^2 of simulated fetal traits from ALSPAC dataset, estimated through conventional GCTA, M-GCTA and H-GCTA approach. Each approach was fitted using GREML (α = -0.25, -1.0), LDAK-Thin (α = -0.25, -1.0) and LDAK-Weights (α = -0.25, -1.0). For GCTA, M is the GRM generated from maternal genotypes (m), and F is the GRM generated from fetal genotypes (f). For M-GCTA, M’ represents the genetic relationship matrix of mothers; G represents genetic relationship matrix of children and D represents mother-child covariance matrix. For H-GCTA, M1 is the GRM generated from maternal transmitted alleles (m1), M2 is the GRM generated from maternal non-transmitted alleles (m2), and P1 is the GRM generated from paternal transmitted alleles (p1). A total of 100 replicates of each phenotype were simulated using empirical genotypes of ALSPAC dataset. P-values were calculated using z test statistics (two sided). (DOCX) [file pgen.1011575.s007.docx]

# **S6 Table: SNP-based heritability of simulated fetal traits from ALSPAC dataset**

| **h^2^ of fetal traits** | | | GREML (alpha = -1.0) | | | GREML (alpha = -0.25) | | | LDAK-Thin (alpha = -1.0) | | | LDAK-Thin (alpha = -0.25) | | | LDAK-Weights (alpha = -1.0) | | | LDAK-Weights (alpha = -0.25) | | |
| --- | --- | --- | --- | --- | --- | --- | --- | --- | --- | --- | --- | --- | --- | --- | --- | --- | --- | --- | --- | --- |
| MAF Cut-off | Approach | GRM | ĥ^2^ | S.E. | p-val | ĥ^2^ | SD | p-val | ĥ^2^ | SD | p-val | ĥ^2^ | SD | p-val | ĥ^2^ | SD | p-val | ĥ^2^ | SD | p-val |
| All Polymorphic SNPs | GCTA | M | 0.0995 | 0.0861 | 2.48E-01 | 0.0583 | 0.0528 | 2.70E-01 | 0.1620 | 0.1575 | 3.04E-01 | 0.0570 | 0.0769 | 4.58E-01 | 0.1841 | 0.2105 | 3.82E-01 | 0.1721 | 0.1529 | 2.60E-01 |
|  |  | F | 0.4569 | 0.0861 | 1.10E-07 | 0.2804 | 0.0528 | 1.11E-07 | 0.6431 | 0.1575 | 4.45E-05 | 0.3451 | 0.0769 | 7.11E-06 | 0.4397 | 0.2105 | 3.67E-02 | 0.4982 | 0.1529 | 1.12E-03 |
|  | M-GCTA | M' | 0.0066 | 0.1125 | 9.54E-01 | 0.0252 | 0.0683 | 7.13E-01 | -0.0459 | 0.1947 | 8.14E-01 | 0.0133 | 0.1004 | 8.95E-01 | -0.2152 | 0.2690 | 4.24E-01 | 0.0825 | 0.1965 | 6.75E-01 |
|  |  | G | 0.4943 | 0.1178 | 2.73E-05 | 0.3251 | 0.0741 | 1.16E-05 | 0.6538 | 0.1730 | 1.58E-04 | 0.3979 | 0.0884 | 6.73E-06 | 0.2971 | 0.2245 | 1.86E-01 | 0.5443 | 0.1819 | 2.77E-03 |
|  |  | D | -0.0407 | 0.0897 | 6.51E-01 | -0.0505 | 0.0562 | 3.68E-01 | 0.0028 | 0.1426 | 9.84E-01 | -0.0560 | 0.0728 | 4.42E-01 | 0.2360 | 0.2016 | 2.42E-01 | -0.0669 | 0.1555 | 6.67E-01 |
|  | H-GCTA | M1 | 0.1964 | 0.0844 | 1.99E-02 | 0.1120 | 0.0540 | 3.79E-02 | 0.2836 | 0.1386 | 4.07E-02 | 0.1287 | 0.0805 | 1.10E-01 | 0.3127 | 0.1890 | 9.80E-02 | 0.2521 | 0.1459 | 8.41E-02 |
|  |  | M2 | 0.0263 | 0.0888 | 7.67E-01 | 0.0274 | 0.0544 | 6.15E-01 | 0.0932 | 0.1349 | 4.90E-01 | 0.0704 | 0.0749 | 3.47E-01 | 0.0260 | 0.2079 | 9.00E-01 | 0.1736 | 0.1445 | 2.30E-01 |
|  |  | P1 | 0.2965 | 0.0880 | 7.58E-04 | 0.2008 | 0.0553 | 2.80E-04 | 0.3394 | 0.1467 | 2.07E-02 | 0.2218 | 0.0734 | 2.51E-03 | 0.0927 | 0.1924 | 6.30E-01 | 0.2537 | 0.1624 | 1.18E-01 |
