# Supplementary material for: Haplotype-based analysis distinguishes maternal-fetal genetic contribution to pregnancy-related outcomes
Source: PLoS Genet. 2025 Mar 10;21(3):e1011575. doi: 10.1371/journal.pgen.1011575 (PMC11918446; doi:10.1371/journal.pgen.1011575)
Supplement: S7 Table — h^2 of simulated traits from ALSPAC dataset with independent maternal-fetal genetic effects (independent sets of causal variants in mother and child), estimated through conventional GCTA, M-GCTA and H-GCTA approach. Each approach was fitted using GREML (α = -0.25, -1.0), LDAK-Thin (α = -0.25, -1.0) and LDAK-Weights (α = -0.25, -1.0). For GCTA, M is the GRM generated from maternal genotypes (m), and F is the GRM generated from fetal genotypes (f). For M-GCTA, M’ represents the genetic relationship matrix of mothers; G represents genetic relationship matrix of children and D represents mother-child covariance matrix. For H-GCTA, M1 is the GRM generated from maternal transmitted alleles (m1), M2 is the GRM generated from maternal non-transmitted alleles (m2), and P1 is the GRM generated from paternal transmitted alleles (p1). A total of 100 replicates of each phenotype were simulated using empirical genotypes of ALSPAC dataset. P-values were calculated using z test statistics (two sided). (DOCX) [file pgen.1011575.s008.docx]

# **S7 Table: SNP-based heritability of simulated traits from ALSPAC dataset with independent maternal-fetal genetic effects using independent sets of causal variants in mother and child**

| **h^2^ of traits with independent maternal-fetal effects (independent sets of causal variants in mothers and fetuses)** | | | GREML (alpha = -1.0) | | | GREML (alpha = -0.25) | | | LDAK-Thin (alpha = -1.0) | | | LDAK-Thin (alpha = -0.25) | | | LDAK-Weights (alpha = -1.0) | | | LDAK-Weights (alpha = -0.25) | | |
| --- | --- | --- | --- | --- | --- | --- | --- | --- | --- | --- | --- | --- | --- | --- | --- | --- | --- | --- | --- | --- |
| MAF Cut-off | Approach | GRM | ĥ^2^ | S.E. | p-val | ĥ^2^ | SD | p-val | ĥ^2^ | SD | p-val | ĥ^2^ | SD | p-val | ĥ^2^ | SD | p-val | ĥ^2^ | SD | p-val |
| All Polymorphic SNPs | GCTA | M | 0.3540 | 0.0905 | 9.20E-05 | 0.1924 | 0.0552 | 4.95E-04 | 0.4911 | 0.1515 | 1.19E-03 | 0.2230 | 0.0771 | 3.81E-03 | 0.5060 | 0.1820 | 5.44E-03 | 0.4078 | 0.1505 | 6.72E-03 |
|  |  | F | 0.3279 | 0.0905 | 2.91E-04 | 0.1795 | 0.0552 | 1.15E-03 | 0.5532 | 0.1515 | 2.60E-04 | 0.2820 | 0.0771 | 2.53E-04 | 0.4846 | 0.1820 | 7.76E-03 | 0.5121 | 0.1505 | 6.65E-04 |
|  | M-GCTA | M' | 0.2224 | 0.1215 | 6.72E-02 | 0.1278 | 0.0761 | 9.30E-02 | 0.2572 | 0.2033 | 2.06E-01 | 0.1322 | 0.1058 | 2.12E-01 | 0.1514 | 0.2587 | 5.58E-01 | 0.1533 | 0.2053 | 4.55E-01 |
|  |  | G | 0.1823 | 0.1052 | 8.31E-02 | 0.1112 | 0.0675 | 9.97E-02 | 0.3497 | 0.1651 | 3.42E-02 | 0.2119 | 0.0869 | 1.47E-02 | 0.1453 | 0.2170 | 5.03E-01 | 0.2762 | 0.1966 | 1.60E-01 |
|  |  | D | 0.0944 | 0.0841 | 2.62E-01 | 0.0418 | 0.0534 | 4.34E-01 | 0.1319 | 0.1255 | 2.93E-01 | 0.0422 | 0.0698 | 5.45E-01 | 0.2626 | 0.1816 | 1.48E-01 | 0.1759 | 0.1482 | 2.35E-01 |
|  | H-GCTA | M1 | 0.2683 | 0.0903 | 2.96E-03 | 0.1225 | 0.0522 | 1.90E-02 | 0.3846 | 0.1388 | 5.59E-03 | 0.1701 | 0.0697 | 1.47E-02 | 0.3947 | 0.1718 | 2.16E-02 | 0.3623 | 0.1453 | 1.26E-02 |
|  |  | M2 | 0.1497 | 0.0948 | 1.14E-01 | 0.0694 | 0.0563 | 2.18E-01 | 0.1975 | 0.1502 | 1.89E-01 | 0.0739 | 0.0831 | 3.74E-01 | 0.1469 | 0.1957 | 4.53E-01 | 0.1066 | 0.1518 | 4.83E-01 |
|  |  | P1 | 0.0878 | 0.0828 | 2.89E-01 | 0.0670 | 0.0525 | 2.02E-01 | 0.1799 | 0.1411 | 2.02E-01 | 0.1336 | 0.0750 | 7.49E-02 | 0.0411 | 0.1732 | 8.13E-01 | 0.1863 | 0.1728 | 2.81E-01 |
