# Supplementary material for: Haplotype-based analysis distinguishes maternal-fetal genetic contribution to pregnancy-related outcomes
Source: PLoS Genet. 2025 Mar 10;21(3):e1011575. doi: 10.1371/journal.pgen.1011575 (PMC11918446; doi:10.1371/journal.pgen.1011575)
Supplement: S8 Table — h^2 of simulated traits from ALSPAC dataset with independent maternal-fetal genetic effects (same set of causal variants in mother and child), estimated through conventional GCTA, M-GCTA and H-GCTA approach. Each approach was fitted using GREML (α = -0.25, -1.0), LDAK-Thin (α = -0.25, -1.0) and LDAK-Weights (α = -0.25, -1.0). For GCTA, M is the GRM generated from maternal genotypes (m), and F is the GRM generated from fetal genotypes (f). For M-GCTA, M’ represents the genetic relationship matrix of mothers; G represents genetic relationship matrix of children and D represents mother-child covariance matrix. For H-GCTA, M1 is the GRM generated from maternal transmitted alleles (m1), M2 is the GRM generated from maternal non-transmitted alleles (m2), and P1 is the GRM generated from paternal transmitted alleles (p1). A total of 100 replicates of each phenotype were simulated using empirical genotypes of ALSPAC dataset. P-values were calculated using z test statistics (two sided). (DOCX) [file pgen.1011575.s009.docx]

# **S8 Table: SNP-based heritability of simulated traits from ALSPAC dataset with independent maternal-fetal genetic effects using same set of causal variants in mother and child**

| **h^2^ of traits with independent maternal-fetal effects (same set of causal variants in mothers and fetuses)** | | | GREML (alpha = -1.0) | | | | | GREML (alpha = -0.25) | | | | | | LDAK-Thin (alpha = -1.0) | | | | | | LDAK-Thin (alpha = -0.25) | | | | | | LDAK-Weights (alpha = -1.0) | | | | | | LDAK-Weights (alpha = -0.25) | | | | | |
| --- | --- | --- | --- | --- | --- | --- | --- | --- | --- | --- | --- | --- | --- | --- | --- | --- | --- | --- | --- | --- | --- | --- | --- | --- | --- | --- | --- | --- | --- | --- | --- | --- | --- | --- | --- | --- | --- |
| MAF Cut-off | Approach | GRM | ĥ^2^ | S.E. | | p-val | | ĥ^2^ | | SD | | p-val | | ĥ^2^ | | SD | | p-val | | ĥ^2^ | | SD | | p-val | | ĥ^2^ | | SD | | p-val | | ĥ^2^ | | SD | | p-val | |
| All Polymorphic SNPs | GCTA | M | 0.3118 | | 0.0961 | | 1.18E-03 | | 0.1580 | | 0.0578 | | 6.21E-03 | | 0.4332 | | 0.1714 | | 1.15E-02 | | 0.1677 | | 0.0825 | | 4.21E-02 | | 0.4239 | | 0.2104 | | 4.40E-02 | | 0.3534 | | 0.1629 | | 3.01E-02 |
|  |  | F | 0.3189 | | 0.0961 | | 9.04E-04 | | 0.1827 | | 0.0578 | | 1.56E-03 | | 0.4268 | | 0.1714 | | 1.28E-02 | | 0.2155 | | 0.0825 | | 9.02E-03 | | 0.3158 | | 0.2104 | | 1.33E-01 | | 0.3514 | | 0.1629 | | 3.10E-02 |
|  | M-GCTA | M' | 0.3050 | | 0.1172 | | 9.24E-03 | | 0.1464 | | 0.0709 | | 3.88E-02 | | 0.3926 | | 0.2099 | | 6.14E-02 | | 0.1582 | | 0.1005 | | 1.15E-01 | | 0.2820 | | 0.2842 | | 3.21E-01 | | 0.2677 | | 0.2250 | | 2.34E-01 |
|  |  | G | 0.3153 | | 0.1248 | | 1.16E-02 | | 0.1799 | | 0.0756 | | 1.74E-02 | | 0.3864 | | 0.1967 | | 4.94E-02 | | 0.2188 | | 0.1031 | | 3.39E-02 | | 0.1431 | | 0.2403 | | 5.51E-01 | | 0.2837 | | 0.1945 | | 1.45E-01 |
|  |  | D | -0.0739 | | 0.0908 | | 4.16E-01 | | -0.0342 | | 0.0553 | | 5.36E-01 | | -0.0667 | | 0.1593 | | 6.75E-01 | | -0.0444 | | 0.0787 | | 5.72E-01 | | 0.0625 | | 0.2143 | | 7.71E-01 | | 0.0043 | | 0.1660 | | 9.79E-01 |
|  | H-GCTA | M1 | 0.2749 | | 0.0941 | | 3.49E-03 | | 0.1597 | | 0.0559 | | 4.26E-03 | | 0.4021 | | 0.1456 | | 5.76E-03 | | 0.1957 | | 0.0782 | | 1.24E-02 | | 0.3115 | | 0.1863 | | 9.45E-02 | | 0.3530 | | 0.1418 | | 1.28E-02 |
|  |  | M2 | 0.1053 | | 0.0976 | | 2.80E-01 | | 0.0455 | | 0.0624 | | 4.66E-01 | | 0.1582 | | 0.1694 | | 3.50E-01 | | 0.0627 | | 0.0839 | | 4.55E-01 | | 0.1235 | | 0.2261 | | 5.85E-01 | | 0.0916 | | 0.1845 | | 6.19E-01 |
|  |  | P1 | 0.1411 | | 0.0801 | | 7.83E-02 | | 0.0691 | | 0.0498 | | 1.65E-01 | | 0.1111 | | 0.1400 | | 4.27E-01 | | 0.0742 | | 0.0765 | | 3.32E-01 | | 0.0158 | | 0.1771 | | 9.29E-01 | | 0.0724 | | 0.1522 | | 6.34E-01 |
