# Supplementary material for: Haplotype-based analysis distinguishes maternal-fetal genetic contribution to pregnancy-related outcomes
Source: PLoS Genet. 2025 Mar 10;21(3):e1011575. doi: 10.1371/journal.pgen.1011575 (PMC11918446; doi:10.1371/journal.pgen.1011575)
Supplement: S9 Table — h^2 of simulated traits with correlated maternal-fetal genetic effects (average correlation = -1.0), estimated through conventional GCTA, M-GCTA and H-GCTA approach. Each approach was fitted using GREML (α = -0.25, -1.0), LDAK-Thin (α = -0.25, -1.0) and LDAK-Weights (α = -0.25, -1.0). For GCTA, M is the GRM generated from maternal genotypes (m), and F is the GRM generated from fetal genotypes (f). For M-GCTA, M’ represents the genetic relationship matrix of mothers; G represents genetic relationship matrix of children and D represents mother-child covariance matrix. For H-GCTA, M1 is the GRM generated from maternal transmitted alleles (m1), M2 is the GRM generated from maternal non-transmitted alleles (m2), and P1 is the GRM generated from paternal transmitted alleles (p1). A total of 100 replicates of each phenotype were simulated using empirical genotypes of ALSPAC dataset. P-values were calculated using z test statistics (two sided). (DOCX) [file pgen.1011575.s010.docx]

# **S9 Table: SNP-based heritability of simulated traits from ALSPAC dataset with correlated maternal-fetal genetic effects (average correlation = -1.0)**

| **h^2^ of traits with correlated maternal-fetal effects (same set of causal variants in mothers and fetuses with average correlation of effects = -1.0)** | | | GREML (alpha = -1.0) | | | | | GREML (alpha = -0.25) | | | | | | LDAK-Thin (alpha = -1.0) | | | | | | LDAK-Thin (alpha = -0.25) | | | | | | LDAK-Weights (alpha = -1.0) | | | | | | LDAK-Weights (alpha = -0.25) | | | | | |
| --- | --- | --- | --- | --- | --- | --- | --- | --- | --- | --- | --- | --- | --- | --- | --- | --- | --- | --- | --- | --- | --- | --- | --- | --- | --- | --- | --- | --- | --- | --- | --- | --- | --- | --- | --- | --- | --- |
| MAF Cut-off | Approach | GRM | ĥ^2^ | S.E. | | p-val | | ĥ^2^ | | SD | | p-val | | ĥ^2^ | | SD | | p-val | | ĥ^2^ | | SD | | p-val | | ĥ^2^ | | SD | | p-val | | ĥ^2^ | | SD | | p-val | |
| All Polymorphic SNPs | GCTA | M | 0.0798 | | 0.1011 | | 4.30E-01 | | 0.0527 | | 0.0624 | | 3.99E-01 | | 0.0985 | | 0.1675 | | 5.56E-01 | | 0.0465 | | 0.0869 | | 5.93E-01 | | 0.0680 | | 0.2071 | | 7.43E-01 | | 0.0483 | | 0.1826 | | 7.91E-01 |
|  |  | F | 0.0753 | | 0.1011 | | 4.57E-01 | | 0.0462 | | 0.0624 | | 4.59E-01 | | 0.0872 | | 0.1675 | | 6.03E-01 | | 0.0562 | | 0.0869 | | 5.18E-01 | | 0.0601 | | 0.2071 | | 7.72E-01 | | 0.0815 | | 0.1826 | | 6.55E-01 |
|  | M-GCTA | M' | 0.3209 | | 0.1204 | | 7.67E-03 | | 0.2067 | | 0.0777 | | 7.76E-03 | | 0.4500 | | 0.1990 | | 2.37E-02 | | 0.2477 | | 0.1071 | | 2.08E-02 | | 0.3000 | | 0.2773 | | 2.79E-01 | | 0.3480 | | 0.2391 | | 1.45E-01 |
|  |  | G | 0.3207 | | 0.1115 | | 4.01E-03 | | 0.2038 | | 0.0738 | | 5.77E-03 | | 0.4488 | | 0.2040 | | 2.78E-02 | | 0.2711 | | 0.1091 | | 1.30E-02 | | 0.2910 | | 0.2719 | | 2.85E-01 | | 0.3943 | | 0.2261 | | 8.12E-02 |
|  |  | D | -0.3271 | | 0.0867 | | 1.61E-04 | | -0.2082 | | 0.0569 | | 2.52E-04 | | -0.4863 | | 0.1687 | | 3.95E-03 | | -0.2723 | | 0.0860 | | 1.54E-03 | | -0.3533 | | 0.2415 | | 1.44E-01 | | -0.4063 | | 0.1907 | | 3.31E-02 |
|  | H-GCTA | M1 | -0.0084 | | 0.1070 | | 9.38E-01 | | -0.0031 | | 0.0679 | | 9.64E-01 | | -0.0274 | | 0.1831 | | 8.81E-01 | | -0.0202 | | 0.0977 | | 8.36E-01 | | 0.0111 | | 0.2125 | | 9.58E-01 | | -0.0022 | | 0.1899 | | 9.91E-01 |
|  |  | M2 | 0.1552 | | 0.0897 | | 8.34E-02 | | 0.1052 | | 0.0582 | | 7.08E-02 | | 0.1970 | | 0.1525 | | 1.96E-01 | | 0.1253 | | 0.0793 | | 1.14E-01 | | 0.0425 | | 0.2245 | | 8.50E-01 | | 0.0889 | | 0.1751 | | 6.12E-01 |
|  |  | P1 | 0.1992 | | 0.0963 | | 3.86E-02 | | 0.1178 | | 0.0612 | | 5.41E-02 | | 0.2887 | | 0.1642 | | 7.86E-02 | | 0.1670 | | 0.0875 | | 5.62E-02 | | 0.1472 | | 0.1983 | | 4.58E-01 | | 0.2404 | | 0.1736 | | 1.66E-01 |
