# Supplementary material for: Haplotype-based analysis distinguishes maternal-fetal genetic contribution to pregnancy-related outcomes
Source: PLoS Genet. 2025 Mar 10;21(3):e1011575. doi: 10.1371/journal.pgen.1011575 (PMC11918446; doi:10.1371/journal.pgen.1011575)
Supplement: S10 Table — h^2 of simulated traits from ALSPAC dataset with correlated maternal-fetal genetic effects (average correlation = -0.5), estimated through conventional GCTA, M-GCTA and H-GCTA approach. Each approach was fitted using GREML (α = -0.25, -1.0), LDAK-Thin (α = -0.25, -1.0) and LDAK-Weights (α = -0.25, -1.0). For GCTA, M is the GRM generated from maternal genotypes (m), and F is the GRM generated from fetal genotypes (f). For M-GCTA, M’ represents the genetic relationship matrix of mothers; G represents genetic relationship matrix of children and D represents mother-child covariance matrix. For H-GCTA, M1 is the GRM generated from maternal transmitted alleles (m1), M2 is the GRM generated from maternal non-transmitted alleles (m2), and P1 is the GRM generated from paternal transmitted alleles (p1). A total of 100 replicates of each phenotype were simulated using empirical genotypes of ALSPAC dataset. P-values were calculated using z test statistics (two sided). (DOCX) [file pgen.1011575.s011.docx]

# **S10 Table: SNP-based heritability of simulated traits from ALSPAC dataset with correlated maternal-fetal genetic effects (average correlation = -0.5)**

| **h^2^ of traits with correlated maternal-fetal effects (same set of causal variants in mothers and fetuses with average correlation of effects = -0.5)** | | | GREML (alpha = -1.0) | | | | | GREML (alpha = -0.25) | | | | | | LDAK-Thin (alpha = -1.0) | | | | | | LDAK-Thin (alpha = -0.25) | | | | | | LDAK-Weights (alpha = -1.0) | | | | | | LDAK-Weights (alpha = -0.25) | | | | | |
| --- | --- | --- | --- | --- | --- | --- | --- | --- | --- | --- | --- | --- | --- | --- | --- | --- | --- | --- | --- | --- | --- | --- | --- | --- | --- | --- | --- | --- | --- | --- | --- | --- | --- | --- | --- | --- | --- |
| MAF Cut-off | Approach | GRM | ĥ^2^ | S.E. | | p-val | | ĥ^2^ | | SD | | p-val | | ĥ^2^ | | SD | | p-val | | ĥ^2^ | | SD | | p-val | | ĥ^2^ | | SD | | p-val | | ĥ^2^ | | SD | | p-val | |
| All Polymorphic SNPs | GCTA | M | 0.2006 | | 0.0893 | | 2.47E-02 | | 0.1325 | | 0.0597 | | 2.65E-02 | | 0.2384 | | 0.1455 | | 1.01E-01 | | 0.1580 | | 0.0826 | | 5.57E-02 | | 0.0880 | | 0.1949 | | 6.52E-01 | | 0.1792 | | 0.1413 | | 2.05E-01 |
|  |  | F | 0.2110 | | 0.0893 | | 1.82E-02 | | 0.1316 | | 0.0597 | | 2.76E-02 | | 0.2366 | | 0.1455 | | 1.04E-01 | | 0.1755 | | 0.0826 | | 3.36E-02 | | -0.0268 | | 0.1949 | | 8.91E-01 | | 0.0891 | | 0.1413 | | 5.28E-01 |
|  | M-GCTA | M' | 0.2442 | | 0.1144 | | 3.29E-02 | | 0.1644 | | 0.0724 | | 2.31E-02 | | 0.3073 | | 0.1967 | | 1.18E-01 | | 0.1925 | | 0.1053 | | 6.76E-02 | | 0.2110 | | 0.2626 | | 4.22E-01 | | 0.2859 | | 0.2046 | | 1.62E-01 |
|  |  | G | 0.2534 | | 0.1175 | | 3.10E-02 | | 0.1634 | | 0.0690 | | 1.78E-02 | | 0.3057 | | 0.2054 | | 1.37E-01 | | 0.2178 | | 0.1040 | | 3.63E-02 | | 0.0021 | | 0.2549 | | 9.93E-01 | | 0.1667 | | 0.1935 | | 3.89E-01 |
|  |  | D | -0.1128 | | 0.0949 | | 2.34E-01 | | -0.0741 | | 0.0594 | | 2.12E-01 | | -0.1661 | | 0.1577 | | 2.92E-01 | | -0.0933 | | 0.0835 | | 2.64E-01 | | -0.1286 | | 0.2218 | | 5.62E-01 | | -0.1595 | | 0.1561 | | 3.07E-01 |
|  | H-GCTA | M1 | 0.1262 | | 0.0958 | | 1.87E-01 | | 0.0970 | | 0.0643 | | 1.31E-01 | | 0.1669 | | 0.1471 | | 2.57E-01 | | 0.1282 | | 0.0921 | | 1.64E-01 | | 0.0584 | | 0.1938 | | 7.63E-01 | | 0.0838 | | 0.1502 | | 5.77E-01 |
|  |  | M2 | 0.1166 | | 0.0910 | | 2.00E-01 | | 0.0738 | | 0.0544 | | 1.75E-01 | | 0.1563 | | 0.1621 | | 3.35E-01 | | 0.0985 | | 0.0748 | | 1.88E-01 | | 0.0600 | | 0.2266 | | 7.91E-01 | | 0.1466 | | 0.1670 | | 3.80E-01 |
|  |  | P1 | 0.1216 | | 0.0889 | | 1.71E-01 | | 0.0704 | | 0.0518 | | 1.74E-01 | | 0.1186 | | 0.1434 | | 4.08E-01 | | 0.0920 | | 0.0740 | | 2.14E-01 | | -0.0458 | | 0.1557 | | 7.69E-01 | | 0.0555 | | 0.1332 | | 6.77E-01 |
