# Supplementary material for: Haplotype-based analysis distinguishes maternal-fetal genetic contribution to pregnancy-related outcomes
Source: PLoS Genet. 2025 Mar 10;21(3):e1011575. doi: 10.1371/journal.pgen.1011575 (PMC11918446; doi:10.1371/journal.pgen.1011575)
Supplement: S11 Table — h^2 of simulated traits from ALSPAC dataset with correlated maternal-fetal genetic effects (average correlation = 1.0), estimated through conventional GCTA, M-GCTA and H-GCTA approach. Each approach was fitted using GREML (α = -0.25, -1.0), LDAK-Thin (α = -0.25, -1.0) and LDAK-Weights (α = -0.25, -1.0). For GCTA, M is the GRM generated from maternal genotypes (m), and F is the GRM generated from fetal genotypes (f). For M-GCTA, M’ represents the genetic relationship matrix of mothers; G represents genetic relationship matrix of children and D represents mother-child covariance matrix. For H-GCTA, M1 is the GRM generated from maternal transmitted alleles (m1), M2 is the GRM generated from maternal non-transmitted alleles (m2), and P1 is the GRM generated from paternal transmitted alleles (p1). A total of 100 replicates of each phenotype were simulated using empirical genotypes of ALSPAC dataset. P-values were calculated using z test statistics (two sided). (DOCX) [file pgen.1011575.s012.docx]

# **S11 Table: SNP-based heritability of simulated traits from ALSPAC dataset with correlated maternal-fetal genetic effects (average correlation = 1.0)**

| **h^2^ of traits with correlated maternal-fetal effects (same set of causal variants in mothers and fetuses with average correlation of effects = 1.0)** | | | GREML (alpha = -1.0) | | | | | GREML (alpha = -0.25) | | | | | | LDAK-Thin (alpha = -1.0) | | | | | | LDAK-Thin (alpha = -0.25) | | | | | | LDAK-Weights (alpha = -1.0) | | | | | | LDAK-Weights (alpha = -0.25) | | | | | |
| --- | --- | --- | --- | --- | --- | --- | --- | --- | --- | --- | --- | --- | --- | --- | --- | --- | --- | --- | --- | --- | --- | --- | --- | --- | --- | --- | --- | --- | --- | --- | --- | --- | --- | --- | --- | --- | --- |
| MAF Cut-off | Approach | GRM | ĥ^2^ | S.E. | | p-val | | ĥ^2^ | | SD | | p-val | | ĥ^2^ | | SD | | p-val | | ĥ^2^ | | SD | | p-val | | ĥ^2^ | | SD | | p-val | | ĥ^2^ | | SD | | p-val | |
| All Polymorphic SNPs | GCTA | M | 0.4836 | | 0.0658 | | 1.99E-13 | | 0.2723 | | 0.0422 | | 1.08E-10 | | 0.7285 | | 0.1181 | | 6.91E-10 | | 0.3378 | | 0.0605 | | 2.34E-08 | | 0.5949 | | 0.1601 | | 2.03E-04 | | 0.5968 | | 0.1263 | | 2.31E-06 |
|  |  | F | 0.4947 | | 0.0658 | | 5.55E-14 | | 0.2863 | | 0.0422 | | 1.14E-11 | | 0.6293 | | 0.1181 | | 9.92E-08 | | 0.3530 | | 0.0605 | | 5.32E-09 | | 0.2954 | | 0.1601 | | 6.51E-02 | | 0.5204 | | 0.1263 | | 3.79E-05 |
|  | M-GCTA | M' | 0.2244 | | 0.1053 | | 3.31E-02 | | 0.1398 | | 0.0660 | | 3.42E-02 | | 0.3949 | | 0.1773 | | 2.59E-02 | | 0.1916 | | 0.0913 | | 3.59E-02 | | 0.3296 | | 0.2255 | | 1.44E-01 | | 0.3814 | | 0.1896 | | 4.43E-02 |
|  |  | G | 0.2479 | | 0.1047 | | 1.79E-02 | | 0.1619 | | 0.0640 | | 1.14E-02 | | 0.2581 | | 0.1791 | | 1.50E-01 | | 0.2159 | | 0.1022 | | 3.46E-02 | | -0.1351 | | 0.2028 | | 5.05E-01 | | 0.2854 | | 0.1893 | | 1.32E-01 |
|  |  | D | 0.1883 | | 0.0837 | | 2.45E-02 | | 0.0906 | | 0.0543 | | 9.55E-02 | | 0.2433 | | 0.1474 | | 9.88E-02 | | 0.0909 | | 0.0821 | | 2.68E-01 | | 0.2952 | | 0.1836 | | 1.08E-01 | | 0.1416 | | 0.1534 | | 3.56E-01 |
|  | H-GCTA | M1 | 0.4399 | | 0.0784 | | 2.01E-08 | | 0.2615 | | 0.0490 | | 9.63E-08 | | 0.6016 | | 0.1448 | | 3.26E-05 | | 0.3258 | | 0.0724 | | 6.78E-06 | | 0.3011 | | 0.1799 | | 9.41E-02 | | 0.5425 | | 0.1376 | | 8.04E-05 |
|  |  | M2 | 0.1201 | | 0.0778 | | 1.23E-01 | | 0.0636 | | 0.0477 | | 1.82E-01 | | 0.1369 | | 0.1299 | | 2.92E-01 | | 0.0501 | | 0.0697 | | 4.72E-01 | | 0.1804 | | 0.1800 | | 3.16E-01 | | 0.0935 | | 0.1381 | | 4.99E-01 |
|  |  | P1 | 0.1093 | | 0.0782 | | 1.62E-01 | | 0.0611 | | 0.0473 | | 1.97E-01 | | 0.0566 | | 0.1251 | | 6.51E-01 | | 0.0436 | | 0.0678 | | 5.20E-01 | | 0.0147 | | 0.1548 | | 9.24E-01 | | 0.0908 | | 0.1214 | | 4.55E-01 |
