# Supplementary material for: Haplotype-based analysis distinguishes maternal-fetal genetic contribution to pregnancy-related outcomes
Source: PLoS Genet. 2025 Mar 10;21(3):e1011575. doi: 10.1371/journal.pgen.1011575 (PMC11918446; doi:10.1371/journal.pgen.1011575)
Supplement: S12 Table — h^2 of simulated traits from ALSPAC dataset with correlated maternal-fetal genetic effects (average correlation = 0.5), estimated through conventional GCTA, M-GCTA and H-GCTA approach. Each approach was fitted using GREML (α = -0.25, -1.0), LDAK-Thin (α = -0.25, -1.0) and LDAK-Weights (α = -0.25, -1.0). For GCTA, M is the GRM generated from maternal genotypes (m), and F is the GRM generated from fetal genotypes (f). For M-GCTA, M’ represents the genetic relationship matrix of mothers; G represents genetic relationship matrix of children and D represents mother-child covariance matrix. For H-GCTA, M1 is the GRM generated from maternal transmitted alleles (m1), M2 is the GRM generated from maternal non-transmitted alleles (m2), and P1 is the GRM generated from paternal transmitted alleles (p1). A total of 100 replicates of each phenotype were simulated using empirical genotypes of ALSPAC dataset. P-values were calculated using z test statistics (two sided). (DOCX) [file pgen.1011575.s013.docx]

# **S12 Table: SNP-based heritability of simulated traits from ALSPAC dataset with correlated maternal-fetal genetic effects (average correlation = 0.5)**

| **h^2^ of traits with correlated maternal-fetal effects (same set of causal variants in mothers and fetuses with average correlation of effects = 0.5)** | | | GREML (alpha = -1.0) | | | | | GREML (alpha = -0.25) | | | | | | LDAK-Thin (alpha = -1.0) | | | | | | LDAK-Thin (alpha = -0.25) | | | | | | LDAK-Weights (alpha = -1.0) | | | | | | LDAK-Weights (alpha = -0.25) | | | | | |
| --- | --- | --- | --- | --- | --- | --- | --- | --- | --- | --- | --- | --- | --- | --- | --- | --- | --- | --- | --- | --- | --- | --- | --- | --- | --- | --- | --- | --- | --- | --- | --- | --- | --- | --- | --- | --- | --- |
| MAF Cut-off | Approach | GRM | ĥ^2^ | S.E. | | p-val | | ĥ^2^ | | SD | | p-val | | ĥ^2^ | | SD | | p-val | | ĥ^2^ | | SD | | p-val | | ĥ^2^ | | SD | | p-val | | ĥ^2^ | | SD | | p-val | |
| All Polymorphic SNPs | GCTA | M | 0.4215 | | 0.0842 | | 5.55E-07 | | 0.2295 | | 0.0545 | | 2.51E-05 | | 0.6160 | | 0.1378 | | 7.77E-06 | | 0.3014 | | 0.0748 | | 5.56E-05 | | 0.3508 | | 0.1980 | | 7.65E-02 | | 0.3892 | | 0.1559 | | 1.25E-02 |
|  |  | F | 0.4009 | | 0.0842 | | 1.92E-06 | | 0.1813 | | 0.0545 | | 8.69E-04 | | 0.6349 | | 0.1378 | | 4.06E-06 | | 0.2465 | | 0.0748 | | 9.77E-04 | | 0.4791 | | 0.1980 | | 1.56E-02 | | 0.4926 | | 0.1559 | | 1.57E-03 |
|  | M-GCTA | M' | 0.2603 | | 0.0984 | | 8.16E-03 | | 0.1500 | | 0.0639 | | 1.90E-02 | | 0.3629 | | 0.1602 | | 2.35E-02 | | 0.2074 | | 0.0907 | | 2.22E-02 | | 0.1037 | | 0.2492 | | 6.77E-01 | | 0.2532 | | 0.1990 | | 2.03E-01 |
|  |  | G | 0.2292 | | 0.1328 | | 8.44E-02 | | 0.0822 | | 0.0826 | | 3.19E-01 | | 0.3669 | | 0.1966 | | 6.20E-02 | | 0.1309 | | 0.1133 | | 2.48E-01 | | 0.2937 | | 0.2302 | | 2.02E-01 | | 0.3810 | | 0.2088 | | 6.80E-02 |
|  |  | D | 0.1022 | | 0.0895 | | 2.54E-01 | | 0.0603 | | 0.0527 | | 2.53E-01 | | 0.1586 | | 0.1441 | | 2.71E-01 | | 0.0667 | | 0.0746 | | 3.71E-01 | | 0.1238 | | 0.2040 | | 5.44E-01 | | 0.0381 | | 0.1558 | | 8.07E-01 |
|  | H-GCTA | M1 | 0.3996 | | 0.0911 | | 1.15E-05 | | 0.2058 | | 0.0523 | | 8.25E-05 | | 0.5761 | | 0.1433 | | 5.78E-05 | | 0.2896 | | 0.0822 | | 4.29E-04 | | 0.2874 | | 0.1780 | | 1.06E-01 | | 0.3860 | | 0.1391 | | 5.51E-03 |
|  |  | M2 | 0.0936 | | 0.0805 | | 2.45E-01 | | 0.0561 | | 0.0508 | | 2.69E-01 | | 0.0512 | | 0.1384 | | 7.11E-01 | | 0.0485 | | 0.0757 | | 5.22E-01 | | -0.0870 | | 0.1962 | | 6.57E-01 | | -0.0002 | | 0.1600 | | 9.99E-01 |
|  |  | P1 | 0.1037 | | 0.0821 | | 2.07E-01 | | 0.0489 | | 0.0536 | | 3.62E-01 | | 0.1769 | | 0.1331 | | 1.84E-01 | | 0.0775 | | 0.0762 | | 3.09E-01 | | 0.1889 | | 0.1790 | | 2.91E-01 | | 0.1873 | | 0.1409 | | 1.84E-01 |
