# Supplementary material for: Haplotype-based analysis distinguishes maternal-fetal genetic contribution to pregnancy-related outcomes
Source: PLoS Genet. 2025 Mar 10;21(3):e1011575. doi: 10.1371/journal.pgen.1011575 (PMC11918446; doi:10.1371/journal.pgen.1011575)
Supplement: S13 Table — h^2 of simulated maternal traits from pooled dataset, estimated through conventional GCTA, M-GCTA and H-GCTA approach. Each approach was fitted using GREML (α = -0.25, -1.0), LDAK-Thin (α = -0.25, -1.0) and LDAK-Weights (α = -0.25, -1.0). For GCTA, M is the GRM generated from maternal genotypes (m), and F is the GRM generated from fetal genotypes (f). For M-GCTA, M’ represents the genetic relationship matrix of mothers; G represents genetic relationship matrix of children and D represents mother-child covariance matrix. For H-GCTA, M1 is the GRM generated from maternal transmitted alleles (m1), M2 is the GRM generated from maternal non-transmitted alleles (m2), and P1 is the GRM generated from paternal transmitted alleles (p1). A total of 100 replicates of each phenotype were simulated using empirical genotypes of Pooled dataset. P-values were calculated using z test statistics (two sided). (DOCX) [file pgen.1011575.s014.docx]

# **S13 Table: SNP-based heritability of simulated maternal traits from Pooled dataset**

| **h^2^ of maternal traits** | | | GREML (alpha = -1.0) | | | GREML (alpha = -0.25) | | | | LDAK-Thin (alpha = -1.0) | | | | LDAK-Thin (alpha = -0.25) | | | | LDAK-Weights (alpha = -1.0) | | | | LDAK-Weights (alpha = -0.25) | | | |  |
| --- | --- | --- | --- | --- | --- | --- | --- | --- | --- | --- | --- | --- | --- | --- | --- | --- | --- | --- | --- | --- | --- | --- | --- | --- | --- | --- |
| MAF Cut-off | Approach | GRM | ĥ^2^ | S.E. | p-val | | ĥ^2^ | SD | p-val | | ĥ^2^ | SD | p-val | | ĥ^2^ | SD | p-val | | ĥ^2^ | SD | p-val | | ĥ^2^ | SD | p-val | |
| All Polymorphic SNPs | GCTA | M | 0.4496 | 0.0857 | 1.53E-07 | | 0.2593 | 0.0543 | 1.83E-06 | | 0.6839 | 0.1416 | 1.36E-06 | | 0.3559 | 0.0730 | 1.09E-06 | | 0.4998 | 0.1991 | 1.21E-02 | | 0.6029 | 0.1490 | 5.19E-05 | |
|  |  | F | 0.1356 | 0.0857 | 1.13E-01 | | 0.0732 | 0.0543 | 1.78E-01 | | 0.1947 | 0.1416 | 1.69E-01 | | 0.0954 | 0.0730 | 1.91E-01 | | 0.1174 | 0.1991 | 5.55E-01 | | 0.1172 | 0.1490 | 4.32E-01 | |
|  | M-GCTA | M' | 0.4056 | 0.0613 | 3.76E-11 | | 0.2534 | 0.0424 | 2.36E-09 | | 0.5351 | 0.0914 | 4.76E-09 | | 0.3243 | 0.0544 | 2.46E-09 | | 0.2660 | 0.1149 | 2.06E-02 | | 0.4369 | 0.1004 | 1.35E-05 | |
|  |  | G | -0.0217 | 0.0604 | 7.20E-01 | | -0.0043 | 0.0377 | 9.09E-01 | | -0.0853 | 0.0990 | 3.89E-01 | | 0.0094 | 0.0514 | 8.55E-01 | | -0.2565 | 0.1313 | 5.08E-02 | | -0.0801 | 0.1079 | 4.58E-01 | |
|  |  | D | 0.0542 | 0.0519 | 2.97E-01 | | 0.0176 | 0.0347 | 6.13E-01 | | 0.1061 | 0.0809 | 1.90E-01 | | 0.0087 | 0.0443 | 8.44E-01 | | 0.2228 | 0.1095 | 4.18E-02 | | 0.0833 | 0.0890 | 3.49E-01 | |
|  | H-GCTA | M1 | 0.2551 | 0.0476 | 8.47E-08 | | 0.1530 | 0.0325 | 2.43E-06 | | 0.3337 | 0.0725 | 4.16E-06 | | 0.1891 | 0.0432 | 1.23E-05 | | 0.2320 | 0.0884 | 8.67E-03 | | 0.2424 | 0.0758 | 1.38E-03 | |
|  |  | M2 | 0.2290 | 0.0446 | 2.92E-07 | | 0.1457 | 0.0294 | 7.12E-07 | | 0.2875 | 0.0692 | 3.21E-05 | | 0.1850 | 0.0396 | 2.91E-06 | | 0.1377 | 0.0840 | 1.01E-01 | | 0.2663 | 0.0667 | 6.55E-05 | |
|  |  | P1 | -0.0305 | 0.0415 | 4.62E-01 | | -0.0146 | 0.0269 | 5.89E-01 | | -0.0713 | 0.0696 | 3.06E-01 | | -0.0058 | 0.0369 | 8.74E-01 | | -0.1923 | 0.0942 | 4.12E-02 | | -0.0766 | 0.0828 | 3.55E-01 | |
