# Supplementary material for: Haplotype-based analysis distinguishes maternal-fetal genetic contribution to pregnancy-related outcomes
Source: PLoS Genet. 2025 Mar 10;21(3):e1011575. doi: 10.1371/journal.pgen.1011575 (PMC11918446; doi:10.1371/journal.pgen.1011575)
Supplement: S14 Table — h^2 of simulated fetal traits from pooled dataset, estimated through conventional GCTA, M-GCTA and H-GCTA approach. Each approach was fitted using GREML (α = -0.25, -1.0), LDAK-Thin (α = -0.25, -1.0) and LDAK-Weights (α = -0.25, -1.0). For GCTA, M is the GRM generated from maternal genotypes (m), and F is the GRM generated from fetal genotypes (f). For M-GCTA, M’ represents the genetic relationship matrix of mothers; G represents genetic relationship matrix of children and D represents mother-child covariance matrix. For H-GCTA, M1 is the GRM generated from maternal transmitted alleles (m1), M2 is the GRM generated from maternal non-transmitted alleles (m2), and P1 is the GRM generated from paternal transmitted alleles (p1). A total of 100 replicates of each phenotype were simulated using empirical genotypes of Pooled dataset. P-values were calculated using z test statistics (two sided). (DOCX) [file pgen.1011575.s015.docx]

# **S14 Table: SNP-based heritability of simulated fetal traits from Pooled dataset**

| **h^2^ of fetal traits** | | | GREML (alpha = -1.0) | | | GREML (alpha = -0.25) | | | LDAK-Thin (alpha = -1.0) | | | LDAK-Thin (alpha = -0.25) | | | LDAK-Weights (alpha = -1.0) | | | LDAK-Weights (alpha = -0.25) | | |
| --- | --- | --- | --- | --- | --- | --- | --- | --- | --- | --- | --- | --- | --- | --- | --- | --- | --- | --- | --- | --- |
| MAF Cut-off | Approach | GRM | ĥ^2^ | S.E. | p-val | ĥ^2^ | SD | p-val | ĥ^2^ | SD | p-val | ĥ^2^ | SD | p-val | ĥ^2^ | SD | p-val | ĥ^2^ | SD | p-val |
| All Polymorphic SNPs | GCTA | M | 0.1087 | 0.0881 | 2.17E-01 | 0.0525 | 0.0533 | 3.24E-01 | 0.2095 | 0.1499 | 1.62E-01 | 0.0645 | 0.0775 | 4.06E-01 | 0.2425 | 0.2067 | 2.41E-01 | 0.2049 | 0.1732 | 2.37E-01 |
|  |  | F | 0.5185 | 0.0881 | 4.03E-09 | 0.3065 | 0.0533 | 8.79E-09 | 0.7035 | 0.1499 | 2.67E-06 | 0.3890 | 0.0775 | 5.20E-07 | 0.4701 | 0.2067 | 2.30E-02 | 0.5476 | 0.1732 | 1.57E-03 |
|  | M-GCTA | M' | -0.0296 | 0.0603 | 6.24E-01 | -0.0089 | 0.0384 | 8.18E-01 | -0.0910 | 0.0974 | 3.50E-01 | -0.0199 | 0.0545 | 7.15E-01 | -0.1236 | 0.1353 | 3.61E-01 | -0.0091 | 0.1135 | 9.36E-01 |
|  |  | G | 0.5265 | 0.0605 | 0.00E+00 | 0.3130 | 0.0366 | 0.00E+00 | 0.7229 | 0.0938 | 1.31E-14 | 0.4025 | 0.0522 | 1.29E-14 | 0.4721 | 0.1264 | 1.88E-04 | 0.5702 | 0.1035 | 3.61E-08 |
|  |  | D | 0.0002 | 0.0469 | 9.96E-01 | -0.0035 | 0.0279 | 8.99E-01 | 0.0094 | 0.0786 | 9.05E-01 | -0.0062 | 0.0402 | 8.77E-01 | 0.0636 | 0.1175 | 5.89E-01 | -0.0117 | 0.0924 | 8.99E-01 |
|  | H-GCTA | M1 | 0.2958 | 0.0430 | 6.35E-12 | 0.1759 | 0.0268 | 5.49E-11 | 0.3923 | 0.0715 | 4.15E-08 | 0.2220 | 0.0386 | 8.86E-09 | 0.2847 | 0.0887 | 1.34E-03 | 0.3217 | 0.0701 | 4.42E-06 |
|  |  | M2 | -0.0355 | 0.0436 | 4.16E-01 | -0.0143 | 0.0271 | 5.96E-01 | -0.0920 | 0.0739 | 2.13E-01 | -0.0251 | 0.0427 | 5.57E-01 | -0.1191 | 0.0920 | 1.96E-01 | -0.0261 | 0.0787 | 7.40E-01 |
|  |  | P1 | 0.2455 | 0.0440 | 2.42E-08 | 0.1379 | 0.0281 | 9.15E-07 | 0.3566 | 0.0672 | 1.09E-07 | 0.1824 | 0.0400 | 5.19E-06 | 0.2880 | 0.0828 | 5.01E-04 | 0.2707 | 0.0676 | 6.22E-05 |
