# Supplementary material for: Haplotype-based analysis distinguishes maternal-fetal genetic contribution to pregnancy-related outcomes
Source: PLoS Genet. 2025 Mar 10;21(3):e1011575. doi: 10.1371/journal.pgen.1011575 (PMC11918446; doi:10.1371/journal.pgen.1011575)
Supplement: S15 Table — h^2 of simulated traits from pooled dataset with independent maternal-fetal genetic effects (independent sets of causal variants in mother and child), estimated through conventional GCTA, M-GCTA and H-GCTA approach. Each approach was fitted using GREML (α = -0.25, -1.0), LDAK-Thin (α = -0.25, -1.0) and LDAK-Weights (α = -0.25, -1.0). For GCTA, M is the GRM generated from maternal genotypes (m), and F is the GRM generated from fetal genotypes (f). For M-GCTA, M’ represents the genetic relationship matrix of mothers; G represents genetic relationship matrix of children and D represents mother-child covariance matrix. For H-GCTA, M1 is the GRM generated from maternal transmitted alleles (m1), M2 is the GRM generated from maternal non-transmitted alleles (m2), and P1 is the GRM generated from paternal transmitted alleles (p1). A total of 100 replicates of each phenotype were simulated using empirical genotypes of Pooled dataset. P-values were calculated using z test statistics (two sided). (DOCX) [file pgen.1011575.s016.docx]

# **S15 Table: SNP-based heritability of simulated traits from Pooled dataset with independent maternal-fetal genetic effects using independent sets of causal variants in mother and child**

| **h^2^ of traits with independent maternal-fetal effects (independent sets of causal variants in mothers and fetuses)** | | | GREML (alpha = -1.0) | | | GREML (alpha = -0.25) | | | LDAK-Thin (alpha = -1.0) | | | LDAK-Thin (alpha = -0.25) | | | LDAK-Weights (alpha = -1.0) | | | LDAK-Weights (alpha = -0.25) | | |
| --- | --- | --- | --- | --- | --- | --- | --- | --- | --- | --- | --- | --- | --- | --- | --- | --- | --- | --- | --- | --- |
| MAF Cut-off | Approach | GRM | ĥ^2^ | S.E. | p-val | ĥ^2^ | SD | p-val | ĥ^2^ | SD | p-val | ĥ^2^ | SD | p-val | ĥ^2^ | SD | p-val | ĥ^2^ | SD | p-val |
| All Polymorphic SNPs | GCTA | M | 0.2489 | 0.0865 | 3.99E-03 | 0.1601 | 0.0539 | 2.98E-03 | 0.3527 | 0.1347 | 8.83E-03 | 0.2146 | 0.0749 | 4.14E-03 | 0.2533 | 0.2021 | 2.10E-01 | 0.3995 | 0.1364 | 3.39E-03 |
|  |  | F | 0.2780 | 0.0865 | 1.30E-03 | 0.1722 | 0.0539 | 1.40E-03 | 0.3259 | 0.1347 | 1.55E-02 | 0.1851 | 0.0749 | 1.34E-02 | 0.1881 | 0.2021 | 3.52E-01 | 0.2754 | 0.1364 | 4.34E-02 |
|  | M-GCTA | M' | 0.2211 | 0.0661 | 8.19E-04 | 0.1361 | 0.0379 | 3.27E-04 | 0.3115 | 0.1068 | 3.52E-03 | 0.1985 | 0.0572 | 5.16E-04 | 0.1642 | 0.1206 | 1.73E-01 | 0.2433 | 0.1014 | 1.64E-02 |
|  |  | G | 0.2681 | 0.0564 | 1.96E-06 | 0.1653 | 0.0356 | 3.42E-06 | 0.2900 | 0.0951 | 2.28E-03 | 0.1834 | 0.0514 | 3.63E-04 | 0.0893 | 0.1268 | 4.81E-01 | 0.2036 | 0.1000 | 4.17E-02 |
|  |  | D | -0.0470 | 0.0554 | 3.97E-01 | -0.0246 | 0.0318 | 4.39E-01 | -0.0692 | 0.0856 | 4.19E-01 | -0.0448 | 0.0438 | 3.06E-01 | 0.0060 | 0.1183 | 9.59E-01 | -0.0010 | 0.0841 | 9.91E-01 |
|  | H-GCTA | M1 | 0.2338 | 0.0460 | 3.72E-07 | 0.1491 | 0.0289 | 2.45E-07 | 0.3024 | 0.0730 | 3.45E-05 | 0.1853 | 0.0413 | 7.33E-06 | 0.2058 | 0.0931 | 2.71E-02 | 0.3042 | 0.0741 | 4.06E-05 |
|  |  | M2 | 0.1238 | 0.0482 | 1.03E-02 | 0.0718 | 0.0294 | 1.48E-02 | 0.1494 | 0.0798 | 6.11E-02 | 0.0984 | 0.0441 | 2.57E-02 | 0.0496 | 0.0918 | 5.89E-01 | 0.0765 | 0.0808 | 3.44E-01 |
|  |  | P1 | 0.1148 | 0.0422 | 6.49E-03 | 0.0754 | 0.0266 | 4.56E-03 | 0.1185 | 0.0691 | 8.65E-02 | 0.0823 | 0.0366 | 2.45E-02 | 0.0091 | 0.0889 | 9.18E-01 | 0.0831 | 0.0749 | 2.67E-01 |
