# Supplementary material for: Haplotype-based analysis distinguishes maternal-fetal genetic contribution to pregnancy-related outcomes
Source: PLoS Genet. 2025 Mar 10;21(3):e1011575. doi: 10.1371/journal.pgen.1011575 (PMC11918446; doi:10.1371/journal.pgen.1011575)
Supplement: S16 Table — h^2 of simulated traits from pooled dataset with independent maternal-fetal genetic effects (same set of causal variants in mother and child), estimated through conventional GCTA, M-GCTA and H-GCTA approach. Each approach was fitted using GREML (α = -0.25, -1.0), LDAK-Thin (α = -0.25, -1.0) and LDAK-Weights (α = -0.25, -1.0). For GCTA, M is the GRM generated from maternal genotypes (m), and F is the GRM generated from fetal genotypes (f). For M-GCTA, M’ represents the genetic relationship matrix of mothers; G represents genetic relationship matrix of children and D represents mother-child covariance matrix. For H-GCTA, M1 is the GRM generated from maternal transmitted alleles (m1), M2 is the GRM generated from maternal non-transmitted alleles (m2), and P1 is the GRM generated from paternal transmitted alleles (p1). A total of 100 replicates of each phenotype were simulated using empirical genotypes of Pooled dataset. P-values were calculated using z test statistics (two sided). (DOCX) [file pgen.1011575.s017.docx]

# **S16 Table: SNP-based heritability of simulated traits from Pooled dataset with independent maternal-fetal genetic effects using same set of causal variants in mother and child**

| **h^2^ of traits with independent maternal-fetal effects (same set of causal variants in mothers and fetuses)** | | | GREML (alpha = -1.0) | | | | | GREML (alpha = -0.25) | | | | | | LDAK-Thin (alpha = -1.0) | | | | | | LDAK-Thin (alpha = -0.25) | | | | | | LDAK-Weights (alpha = -1.0) | | | | | | LDAK-Weights (alpha = -0.25) | | | | | |
| --- | --- | --- | --- | --- | --- | --- | --- | --- | --- | --- | --- | --- | --- | --- | --- | --- | --- | --- | --- | --- | --- | --- | --- | --- | --- | --- | --- | --- | --- | --- | --- | --- | --- | --- | --- | --- | --- |
| MAF Cut-off | Approach | GRM | ĥ^2^ | S.E. | | p-val | | ĥ^2^ | | SD | | p-val | | ĥ^2^ | | SD | | p-val | | ĥ^2^ | | SD | | p-val | | ĥ^2^ | | SD | | p-val | | ĥ^2^ | | SD | | p-val | |
| All Polymorphic SNPs | GCTA | M | 0.3387 | | 0.0899 | | 1.64E-04 | | 0.2085 | | 0.0574 | | 2.78E-04 | | 0.4479 | | 0.1445 | | 1.93E-03 | | 0.2546 | | 0.0776 | | 1.03E-03 | | 0.2751 | | 0.2150 | | 2.01E-01 | | 0.3336 | | 0.1487 | | 2.49E-02 |
|  |  | F | 0.2698 | | 0.0899 | | 2.67E-03 | | 0.1598 | | 0.0574 | | 5.34E-03 | | 0.3301 | | 0.1445 | | 2.23E-02 | | 0.1804 | | 0.0776 | | 2.01E-02 | | 0.2603 | | 0.2150 | | 2.26E-01 | | 0.2950 | | 0.1487 | | 4.73E-02 |
|  | M-GCTA | M' | 0.2933 | | 0.0627 | | 2.88E-06 | | 0.1938 | | 0.0395 | | 9.12E-07 | | 0.3824 | | 0.1024 | | 1.88E-04 | | 0.2499 | | 0.0548 | | 5.14E-06 | | 0.1734 | | 0.1295 | | 1.81E-01 | | 0.3876 | | 0.1127 | | 5.84E-04 |
|  |  | G | 0.2163 | | 0.0596 | | 2.86E-04 | | 0.1322 | | 0.0390 | | 6.97E-04 | | 0.2258 | | 0.0950 | | 1.75E-02 | | 0.1550 | | 0.0532 | | 3.59E-03 | | 0.1080 | | 0.1281 | | 3.99E-01 | | 0.2626 | | 0.1008 | | 9.22E-03 |
|  |  | D | -0.0291 | | 0.0502 | | 5.62E-01 | | -0.0261 | | 0.0333 | | 4.33E-01 | | -0.0369 | | 0.0820 | | 6.52E-01 | | -0.0455 | | 0.0452 | | 3.14E-01 | | 0.0383 | | 0.1033 | | 7.11E-01 | | -0.0949 | | 0.0914 | | 2.99E-01 |
|  | H-GCTA | M1 | 0.2368 | | 0.0463 | | 3.24E-07 | | 0.1470 | | 0.0300 | | 9.79E-07 | | 0.2862 | | 0.0701 | | 4.47E-05 | | 0.1690 | | 0.0404 | | 2.84E-05 | | 0.1791 | | 0.0920 | | 5.16E-02 | | 0.2279 | | 0.0697 | | 1.07E-03 |
|  |  | M2 | 0.1522 | | 0.0416 | | 2.52E-04 | | 0.0965 | | 0.0273 | | 4.01E-04 | | 0.1720 | | 0.0780 | | 2.74E-02 | | 0.1283 | | 0.0377 | | 6.60E-04 | | 0.0401 | | 0.1103 | | 7.16E-01 | | 0.1801 | | 0.0867 | | 3.77E-02 |
|  |  | P1 | 0.1117 | | 0.0421 | | 7.92E-03 | | 0.0672 | | 0.0270 | | 1.26E-02 | | 0.1539 | | 0.0708 | | 2.96E-02 | | 0.0954 | | 0.0407 | | 1.91E-02 | | 0.1310 | | 0.0933 | | 1.60E-01 | | 0.2131 | | 0.0773 | | 5.86E-03 |
