# Supplementary material for: Haplotype-based analysis distinguishes maternal-fetal genetic contribution to pregnancy-related outcomes
Source: PLoS Genet. 2025 Mar 10;21(3):e1011575. doi: 10.1371/journal.pgen.1011575 (PMC11918446; doi:10.1371/journal.pgen.1011575)
Supplement: S17 Table — h^2 of simulated traits from pooled dataset with correlated maternal-fetal genetic effects (average correlation = -1.0), estimated through conventional GCTA, M-GCTA and H-GCTA approach. Each approach was fitted using GREML (α = -0.25, -1.0), LDAK-Thin (α = -0.25, -1.0) and LDAK-Weights (α = -0.25, -1.0). For GCTA, M is the GRM generated from maternal genotypes (m), and F is the GRM generated from fetal genotypes (f). For M-GCTA, M’ represents the genetic relationship matrix of mothers; G represents genetic relationship matrix of children and D represents mother-child covariance matrix. For H-GCTA, M1 is the GRM generated from maternal transmitted alleles (m1), M2 is the GRM generated from maternal non-transmitted alleles (m2), and P1 is the GRM generated from paternal transmitted alleles (p1). A total of 100 replicates of each phenotype were simulated using empirical genotypes of Pooled dataset. P-values were calculated using z test statistics (two sided). (DOCX) [file pgen.1011575.s018.docx]

# **S17 Table: SNP-based heritability of simulated traits from Pooled dataset with correlated maternal-fetal genetic effects (average correlation = -1.0)**

| **h^2^ of traits with correlated maternal-fetal effects (same set of causal variants in mothers and fetuses with average correlation of effects = -1.0)** | | | GREML (alpha = -1.0) | | | | | GREML (alpha = -0.25) | | | | | | LDAK-Thin (alpha = -1.0) | | | | | | LDAK-Thin (alpha = -0.25) | | | | | | LDAK-Weights (alpha = -1.0) | | | | | | LDAK-Weights (alpha = -0.25) | | | | | |
| --- | --- | --- | --- | --- | --- | --- | --- | --- | --- | --- | --- | --- | --- | --- | --- | --- | --- | --- | --- | --- | --- | --- | --- | --- | --- | --- | --- | --- | --- | --- | --- | --- | --- | --- | --- | --- | --- |
| MAF Cut-off | Approach | GRM | ĥ^2^ | S.E. | | p-val | | ĥ^2^ | | SD | | p-val | | ĥ^2^ | | SD | | p-val | | ĥ^2^ | | SD | | p-val | | ĥ^2^ | | SD | | p-val | | ĥ^2^ | | SD | | p-val | |
| All Polymorphic SNPs | GCTA | M | 0.0821 | | 0.0938 | | 3.82E-01 | | 0.0529 | | 0.0596 | | 3.74E-01 | | 0.1398 | | 0.1515 | | 3.56E-01 | | 0.0789 | | 0.0872 | | 3.65E-01 | | 0.0894 | | 0.1684 | | 5.95E-01 | | 0.0839 | | 0.1504 | | 5.77E-01 |
|  |  | F | 0.1142 | | 0.0938 | | 2.24E-01 | | 0.0617 | | 0.0596 | | 3.00E-01 | | 0.1620 | | 0.1515 | | 2.85E-01 | | 0.0732 | | 0.0872 | | 4.01E-01 | | 0.1261 | | 0.1684 | | 4.54E-01 | | 0.1019 | | 0.1504 | | 4.98E-01 |
|  | M-GCTA | M' | 0.3587 | | 0.0835 | | 1.74E-05 | | 0.2121 | | 0.0432 | | 8.91E-07 | | 0.4642 | | 0.1065 | | 1.31E-05 | | 0.2676 | | 0.0548 | | 1.05E-06 | | 0.3115 | | 0.1419 | | 2.81E-02 | | 0.4330 | | 0.1035 | | 2.87E-05 |
|  |  | G | 0.3826 | | 0.0678 | | 1.70E-08 | | 0.2326 | | 0.0419 | | 2.91E-08 | | 0.5245 | | 0.1100 | | 1.87E-06 | | 0.2891 | | 0.0555 | | 1.93E-07 | | 0.3698 | | 0.1425 | | 9.44E-03 | | 0.4379 | | 0.1071 | | 4.34E-05 |
|  |  | D | -0.3609 | | 0.0624 | | 7.10E-09 | | -0.2281 | | 0.0331 | | 5.92E-12 | | -0.5161 | | 0.0794 | | 8.20E-11 | | -0.2867 | | 0.0445 | | 1.20E-10 | | -0.3707 | | 0.1035 | | 3.42E-04 | | -0.4591 | | 0.0825 | | 2.60E-08 |
|  | H-GCTA | M1 | -0.0101 | | 0.0504 | | 8.41E-01 | | -0.0018 | | 0.0317 | | 9.55E-01 | | -0.0433 | | 0.0812 | | 5.94E-01 | | -0.0023 | | 0.0445 | | 9.58E-01 | | -0.0755 | | 0.1114 | | 4.98E-01 | | -0.0412 | | 0.0938 | | 6.60E-01 |
|  |  | M2 | 0.1757 | | 0.0524 | | 7.92E-04 | | 0.1006 | | 0.0312 | | 1.24E-03 | | 0.2425 | | 0.0852 | | 4.45E-03 | | 0.1356 | | 0.0414 | | 1.04E-03 | | 0.1688 | | 0.1176 | | 1.51E-01 | | 0.2240 | | 0.0905 | | 1.33E-02 |
|  |  | P1 | 0.2007 | | 0.0405 | | 7.32E-07 | | 0.1124 | | 0.0249 | | 6.18E-06 | | 0.2680 | | 0.0645 | | 3.23E-05 | | 0.1409 | | 0.0331 | | 2.09E-05 | | 0.1646 | | 0.1009 | | 1.03E-01 | | 0.2190 | | 0.0803 | | 6.40E-03 |
