# Supplementary material for: Haplotype-based analysis distinguishes maternal-fetal genetic contribution to pregnancy-related outcomes
Source: PLoS Genet. 2025 Mar 10;21(3):e1011575. doi: 10.1371/journal.pgen.1011575 (PMC11918446; doi:10.1371/journal.pgen.1011575)
Supplement: S18 Table — h^2 of simulated traits from pooled dataset with correlated maternal-fetal genetic effects (average correlation = -0.5), estimated through conventional GCTA, M-GCTA and H-GCTA approach. Each approach was fitted using GREML (α = -0.25, -1.0), LDAK-Thin (α = -0.25, -1.0) and LDAK-Weights (α = -0.25, -1.0). For GCTA, M is the GRM generated from maternal genotypes (m), and F is the GRM generated from fetal genotypes (f). For M-GCTA, M’ represents the genetic relationship matrix of mothers; G represents genetic relationship matrix of children and D represents mother-child covariance matrix. For H-GCTA, M1 is the GRM generated from maternal transmitted alleles (m1), M2 is the GRM generated from maternal non-transmitted alleles (m2), and P1 is the GRM generated from paternal transmitted alleles (p1). A total of 100 replicates of each phenotype were simulated using empirical genotypes of Pooled dataset. P-values were calculated using z test statistics (two sided). (DOCX) [file pgen.1011575.s019.docx]

# **S18 Table: SNP-based heritability of simulated traits from Pooled dataset with correlated maternal-fetal genetic effects (average correlation = -0.5)**

| **h^2^ of traits with correlated maternal-fetal effects (same set of causal variants in mothers and fetuses with average correlation of effects = -0.5)** | | | GREML (alpha = -1.0) | | | | | GREML (alpha = -0.25) | | | | | | LDAK-Thin (alpha = -1.0) | | | | | | LDAK-Thin (alpha = -0.25) | | | | | | LDAK-Weights (alpha = -1.0) | | | | | | LDAK-Weights (alpha = -0.25) | | | | | |
| --- | --- | --- | --- | --- | --- | --- | --- | --- | --- | --- | --- | --- | --- | --- | --- | --- | --- | --- | --- | --- | --- | --- | --- | --- | --- | --- | --- | --- | --- | --- | --- | --- | --- | --- | --- | --- | --- |
| MAF Cut-off | Approach | GRM | ĥ^2^ | S.E. | | p-val | | ĥ^2^ | | SD | | p-val | | ĥ^2^ | | SD | | p-val | | ĥ^2^ | | SD | | p-val | | ĥ^2^ | | SD | | p-val | | ĥ^2^ | | SD | | p-val | |
| All Polymorphic SNPs | GCTA | M | 0.1683 | | 0.0899 | | 6.11E-02 | | 0.0928 | | 0.0586 | | 1.13E-01 | | 0.2337 | | 0.1562 | | 1.35E-01 | | 0.1342 | | 0.0817 | | 1.00E-01 | | 0.1249 | | 0.2185 | | 5.68E-01 | | 0.1780 | | 0.1584 | | 2.61E-01 |
|  |  | F | 0.2373 | | 0.0899 | | 8.27E-03 | | 0.1448 | | 0.0586 | | 1.35E-02 | | 0.3062 | | 0.1562 | | 5.00E-02 | | 0.1689 | | 0.0817 | | 3.86E-02 | | 0.2885 | | 0.2185 | | 1.87E-01 | | 0.2861 | | 0.1584 | | 7.09E-02 |
|  | M-GCTA | M' | 0.2617 | | 0.0595 | | 1.10E-05 | | 0.1521 | | 0.0402 | | 1.53E-04 | | 0.3462 | | 0.0954 | | 2.83E-04 | | 0.2042 | | 0.0567 | | 3.15E-04 | | 0.1689 | | 0.1165 | | 1.47E-01 | | 0.3211 | | 0.1009 | | 1.47E-03 |
|  |  | G | 0.2956 | | 0.0624 | | 2.16E-06 | | 0.1675 | | 0.0396 | | 2.33E-05 | | 0.3561 | | 0.1052 | | 7.09E-04 | | 0.1952 | | 0.0605 | | 1.25E-03 | | 0.2444 | | 0.1295 | | 5.92E-02 | | 0.3231 | | 0.1036 | | 1.82E-03 |
|  |  | D | -0.1359 | | 0.0501 | | 6.69E-03 | | -0.0684 | | 0.0331 | | 3.89E-02 | | -0.1782 | | 0.0824 | | 3.06E-02 | | -0.0908 | | 0.0463 | | 5.00E-02 | | -0.0575 | | 0.0949 | | 5.44E-01 | | -0.1545 | | 0.0848 | | 6.85E-02 |
|  | H-GCTA | M1 | 0.1346 | | 0.0405 | | 9.02E-04 | | 0.0925 | | 0.0277 | | 8.47E-04 | | 0.1393 | | 0.0675 | | 3.89E-02 | | 0.0956 | | 0.0391 | | 1.45E-02 | | 0.0767 | | 0.0906 | | 3.97E-01 | | 0.1084 | | 0.0690 | | 1.16E-01 |
|  |  | M2 | 0.1275 | | 0.0432 | | 3.16E-03 | | 0.0761 | | 0.0289 | | 8.36E-03 | | 0.1847 | | 0.0606 | | 2.31E-03 | | 0.1115 | | 0.0355 | | 1.71E-03 | | 0.1072 | | 0.0862 | | 2.14E-01 | | 0.1962 | | 0.0699 | | 4.98E-03 |
|  |  | P1 | 0.1473 | | 0.0441 | | 8.31E-04 | | 0.0840 | | 0.0268 | | 1.76E-03 | | 0.1792 | | 0.0723 | | 1.32E-02 | | 0.0963 | | 0.0410 | | 1.90E-02 | | 0.1880 | | 0.0878 | | 3.22E-02 | | 0.1849 | | 0.0690 | | 7.40E-03 |
