# Supplementary material for: Haplotype-based analysis distinguishes maternal-fetal genetic contribution to pregnancy-related outcomes
Source: PLoS Genet. 2025 Mar 10;21(3):e1011575. doi: 10.1371/journal.pgen.1011575 (PMC11918446; doi:10.1371/journal.pgen.1011575)
Supplement: S19 Table — h^2 of simulated traits from pooled dataset with correlated maternal-fetal genetic effects (average correlation = 1.0), estimated through conventional GCTA, M-GCTA and H-GCTA approach. Each approach was fitted using GREML (α = -0.25, -1.0), LDAK-Thin (α = -0.25, -1.0) and LDAK-Weights (α = -0.25, -1.0). For GCTA, M is the GRM generated from maternal genotypes (m), and F is the GRM generated from fetal genotypes (f). For M-GCTA, M’ represents the genetic relationship matrix of mothers; G represents genetic relationship matrix of children and D represents mother-child covariance matrix. For H-GCTA, M1 is the GRM generated from maternal transmitted alleles (m1), M2 is the GRM generated from maternal non-transmitted alleles (m2), and P1 is the GRM generated from paternal transmitted alleles (p1). A total of 100 replicates of each phenotype were simulated using empirical genotypes of Pooled dataset. P-values were calculated using z test statistics (two sided). (DOCX) [file pgen.1011575.s020.docx]

# **S19 Table: SNP-based heritability of simulated traits from Pooled dataset with correlated maternal-fetal genetic effects (average correlation = 1.0)**

| **h^2^ of traits with correlated maternal-fetal effects (same set of causal variants in mothers and fetuses with average correlation of effects = 1.0)** | | | GREML (alpha = -1.0) | | | | | GREML (alpha = -0.25) | | | | | | LDAK-Thin (alpha = -1.0) | | | | | | LDAK-Thin (alpha = -0.25) | | | | | | LDAK-Weights (alpha = -1.0) | | | | | | LDAK-Weights (alpha = -0.25) | | | | | |
| --- | --- | --- | --- | --- | --- | --- | --- | --- | --- | --- | --- | --- | --- | --- | --- | --- | --- | --- | --- | --- | --- | --- | --- | --- | --- | --- | --- | --- | --- | --- | --- | --- | --- | --- | --- | --- | --- |
| MAF Cut-off | Approach | GRM | ĥ^2^ | S.E. | | p-val | | ĥ^2^ | | SD | | p-val | | ĥ^2^ | | SD | | p-val | | ĥ^2^ | | SD | | p-val | | ĥ^2^ | | SD | | p-val | | ĥ^2^ | | SD | | p-val | |
| All Polymorphic SNPs | GCTA | M | 0.4622 | | 0.0855 | | 6.41E-08 | | 0.2519 | | 0.0537 | | 2.73E-06 | | 0.5912 | | 0.1444 | | 4.22E-05 | | 0.2638 | | 0.0775 | | 6.62E-04 | | 0.5531 | | 0.1819 | | 2.35E-03 | | 0.4885 | | 0.1572 | | 1.89E-03 |
|  |  | F | 0.4475 | | 0.0855 | | 1.65E-07 | | 0.2845 | | 0.0537 | | 1.18E-07 | | 0.6013 | | 0.1444 | | 3.11E-05 | | 0.3508 | | 0.0775 | | 5.94E-06 | | 0.3614 | | 0.1819 | | 4.69E-02 | | 0.4774 | | 0.1572 | | 2.39E-03 |
|  | M-GCTA | M' | 0.1885 | | 0.0501 | | 1.71E-04 | | 0.1088 | | 0.0310 | | 4.56E-04 | | 0.1716 | | 0.0744 | | 2.10E-02 | | 0.1245 | | 0.0411 | | 2.44E-03 | | 0.0336 | | 0.0998 | | 7.36E-01 | | 0.1504 | | 0.0959 | | 1.17E-01 |
|  |  | G | 0.2059 | | 0.0560 | | 2.39E-04 | | 0.1536 | | 0.0342 | | 7.15E-06 | | 0.2440 | | 0.0902 | | 6.84E-03 | | 0.1942 | | 0.0493 | | 8.07E-05 | | 0.0271 | | 0.1234 | | 8.26E-01 | | 0.1942 | | 0.1024 | | 5.79E-02 |
|  |  | D | 0.1968 | | 0.0459 | | 1.83E-05 | | 0.1084 | | 0.0278 | | 9.82E-05 | | 0.3004 | | 0.0705 | | 2.02E-05 | | 0.1306 | | 0.0382 | | 6.30E-04 | | 0.3017 | | 0.0859 | | 4.43E-04 | | 0.2293 | | 0.0749 | | 2.20E-03 |
|  | H-GCTA | M1 | 0.3744 | | 0.0419 | | 0.00E+00 | | 0.2266 | | 0.0261 | | 0.00E+00 | | 0.4750 | | 0.0634 | | 6.46E-14 | | 0.2727 | | 0.0350 | | 6.22E-15 | | 0.2879 | | 0.0822 | | 4.61E-04 | | 0.4028 | | 0.0690 | | 5.26E-09 |
|  |  | M2 | 0.1235 | | 0.0374 | | 9.67E-04 | | 0.0731 | | 0.0231 | | 1.52E-03 | | 0.1289 | | 0.0522 | | 1.35E-02 | | 0.0950 | | 0.0323 | | 3.26E-03 | | 0.0432 | | 0.0735 | | 5.56E-01 | | 0.1414 | | 0.0598 | | 1.80E-02 |
|  |  | P1 | 0.1106 | | 0.0390 | | 4.58E-03 | | 0.0798 | | 0.0248 | | 1.30E-03 | | 0.1180 | | 0.0646 | | 6.77E-02 | | 0.0943 | | 0.0348 | | 6.80E-03 | | 0.0491 | | 0.0870 | | 5.73E-01 | | 0.0904 | | 0.0750 | | 2.28E-01 |
