# Supplementary material for: Haplotype-based analysis distinguishes maternal-fetal genetic contribution to pregnancy-related outcomes
Source: PLoS Genet. 2025 Mar 10;21(3):e1011575. doi: 10.1371/journal.pgen.1011575 (PMC11918446; doi:10.1371/journal.pgen.1011575)
Supplement: S20 Table — h^2 of simulated traits from pooled dataset with correlated maternal-fetal genetic effects (average correlation = 0.5), estimated through conventional GCTA, M-GCTA and H-GCTA approach. Each approach was fitted using GREML (α = -0.25, -1.0), LDAK-Thin (α = -0.25, -1.0) and LDAK-Weights (α = -0.25, -1.0). For GCTA, M is the GRM generated from maternal genotypes (m), and F is the GRM generated from fetal genotypes (f). For M-GCTA, M’ represents the genetic relationship matrix of mothers; G represents genetic relationship matrix of children and D represents mother-child covariance matrix. For H-GCTA, M1 is the GRM generated from maternal transmitted alleles (m1), M2 is the GRM generated from maternal non-transmitted alleles (m2), and P1 is the GRM generated from paternal transmitted alleles (p1). A total of 100 replicates of each phenotype were simulated using empirical genotypes of Pooled dataset. P-values were calculated using z test statistics (two sided). (DOCX) [file pgen.1011575.s021.docx]

# **S20 Table: SNP-based heritability of simulated traits from Pooled dataset with correlated maternal-fetal genetic effects (average correlation = 0.5)**

| **h^2^ of traits with correlated maternal-fetal effects (same set of causal variants in mothers and fetuses with average correlation of effects = 0.5)** | | | GREML (alpha = -1.0) | | | | | GREML (alpha = -0.25) | | | | | | LDAK-Thin (alpha = -1.0) | | | | | | LDAK-Thin (alpha = -0.25) | | | | | | LDAK-Weights (alpha = -1.0) | | | | | | LDAK-Weights (alpha = -0.25) | | | | | |
| --- | --- | --- | --- | --- | --- | --- | --- | --- | --- | --- | --- | --- | --- | --- | --- | --- | --- | --- | --- | --- | --- | --- | --- | --- | --- | --- | --- | --- | --- | --- | --- | --- | --- | --- | --- | --- | --- |
| MAF Cut-off | Approach | GRM | ĥ^2^ | S.E. | | p-val | | ĥ^2^ | | SD | | p-val | | ĥ^2^ | | SD | | p-val | | ĥ^2^ | | SD | | p-val | | ĥ^2^ | | SD | | p-val | | ĥ^2^ | | SD | | p-val | |
| All Polymorphic SNPs | GCTA | M | 0.4323 | | 0.0697 | | 5.60E-10 | | 0.2431 | | 0.0489 | | 6.53E-07 | | 0.5340 | | 0.1180 | | 6.07E-06 | | 0.2692 | | 0.0755 | | 3.63E-04 | | 0.4577 | | 0.1742 | | 8.58E-03 | | 0.3567 | | 0.1234 | | 3.84E-03 |
|  |  | F | 0.4100 | | 0.0697 | | 4.10E-09 | | 0.2422 | | 0.0489 | | 7.19E-07 | | 0.5348 | | 0.1180 | | 5.88E-06 | | 0.2973 | | 0.0755 | | 8.21E-05 | | 0.3993 | | 0.1742 | | 2.19E-02 | | 0.4307 | | 0.1234 | | 4.82E-04 |
|  | M-GCTA | M' | 0.2727 | | 0.0549 | | 6.87E-07 | | 0.1680 | | 0.0373 | | 6.84E-06 | | 0.3224 | | 0.0912 | | 4.08E-04 | | 0.1904 | | 0.0536 | | 3.84E-04 | | 0.2307 | | 0.1102 | | 3.64E-02 | | 0.2407 | | 0.1028 | | 1.92E-02 |
|  |  | G | 0.2128 | | 0.0559 | | 1.39E-04 | | 0.1165 | | 0.0377 | | 2.00E-03 | | 0.2353 | | 0.0845 | | 5.38E-03 | | 0.1459 | | 0.0477 | | 2.21E-03 | | 0.0645 | | 0.1056 | | 5.42E-01 | | 0.1686 | | 0.0979 | | 8.52E-02 |
|  |  | D | 0.1020 | | 0.0462 | | 2.73E-02 | | 0.0674 | | 0.0301 | | 2.54E-02 | | 0.1618 | | 0.0753 | | 3.15E-02 | | 0.0836 | | 0.0403 | | 3.79E-02 | | 0.1559 | | 0.0933 | | 9.48E-02 | | 0.1577 | | 0.0777 | | 4.25E-02 |
|  | H-GCTA | M1 | 0.3465 | | 0.0366 | | 0.00E+00 | | 0.2180 | | 0.0240 | | 0.00E+00 | | 0.4684 | | 0.0652 | | 6.63E-13 | | 0.2751 | | 0.0360 | | 2.20E-14 | | 0.3384 | | 0.0936 | | 3.01E-04 | | 0.4189 | | 0.0768 | | 4.84E-08 |
|  |  | M2 | 0.1438 | | 0.0388 | | 2.11E-04 | | 0.0833 | | 0.0238 | | 4.63E-04 | | 0.1579 | | 0.0650 | | 1.51E-02 | | 0.0933 | | 0.0339 | | 5.94E-03 | | 0.1270 | | 0.0921 | | 1.68E-01 | | 0.1262 | | 0.0732 | | 8.47E-02 |
|  |  | P1 | 0.1154 | | 0.0424 | | 6.50E-03 | | 0.0676 | | 0.0276 | | 1.43E-02 | | 0.1030 | | 0.0652 | | 1.14E-01 | | 0.0856 | | 0.0377 | | 2.30E-02 | | 0.0103 | | 0.0755 | | 8.92E-01 | | 0.0710 | | 0.0725 | | 3.27E-01 |
