# Supplementary material for: Haplotype-based analysis distinguishes maternal-fetal genetic contribution to pregnancy-related outcomes
Source: PLoS Genet. 2025 Mar 10;21(3):e1011575. doi: 10.1371/journal.pgen.1011575 (PMC11918446; doi:10.1371/journal.pgen.1011575)
Supplement: S21 Table — h^2 of simulated fetal traits with POEs from pooled dataset, estimated through conventional GCTA, M-GCTA and H-GCTA approach using GREML (α = -1.0) model – A) 50% causal variants with POEs; B) 25% causal variants with POEs. POEs were incorporated by reducing the effect of m1 as compared to p1 by multiplying effects of m1 with (1 – I) where I is the imprinting factor such as 0.25, 0.50, 0.75 and 1.0. In each scenario, m1 shows either no imprinting, i.e., I = 0.0 (m1/p1=1.0/1.0) or partial imprinting, i.e., I = 0.25-0.75 (m1/p1=0.75/1.0−0.25/1.0) or complete imprinting, i.e., I = 1.0 (m1/p1=0.0/1.0). For GCTA, M is the GRM generated from maternal genotypes (m), and F is the GRM generated from fetal genotypes (f). For M-GCTA, M’ represents the genetic relationship matrix of mothers; G represents genetic relationship matrix of children and D represents mother-child covariance matrix. For H-GCTA, M1 is the GRM generated from maternal transmitted alleles (m1), M2 is the GRM generated from maternal non-transmitted alleles (m2), and P1 is the GRM generated from paternal transmitted alleles (p1). A total of 100 replicates of each phenotype were simulated using empirical genotypes of Pooled dataset. P-values were calculated using z test statistics (two sided). (DOCX) [file pgen.1011575.s022.docx]

# **S21 Table: SNP-based heritability of simulated fetal traits with parent-of-origin effects (POEs) from Pooled dataset**

| **50% variants with POEs** |  |  |  |  |  |  |  |  |  |  |  |  |  |  |  |  |
| --- | --- | --- | --- | --- | --- | --- | --- | --- | --- | --- | --- | --- | --- | --- | --- | --- |
| **Approach** | **GRM** | **m1/p1 = (1.0/1.0)** | | | **m1/p1 = (0.75/1.0)** | | | **m1/p1 = (0.50/1.0)** | | | **m1/p1 = (0.25/1.0)** | | | **m1/p1 = (0.0/1.0)** | | |
|  |  | **ĥ^2^** | **S.E.** | **p-val** | **ĥ^2^** | **S.E.** | **p-val** | **ĥ^2^** | **S.E.** | **p-val** | **ĥ^2^** | **S.E.** | **p-val** | **ĥ^2^** | **S.E.** | **p-val** |
| GCTA | M | 0.1139 | 0.0904 | 2.08E-01 | 0.1387 | 0.0830 | 9.47E-02 | 0.0619 | 0.0820 | 4.50E-01 | 0.0626 | 0.0828 | 4.50E-01 | 0.0093 | 0.0844 | 9.12E-01 |
|  | F | 0.5110 | 0.0904 | 1.60E-08 | 0.4812 | 0.0830 | 6.81E-09 | 0.4979 | 0.0820 | 1.24E-09 | 0.4481 | 0.0828 | 6.32E-08 | 0.4210 | 0.0844 | 6.11E-07 |
| M-GCTA | M' | -0.0477 | 0.0564 | 3.98E-01 | 0.0318 | 0.0492 | 5.18E-01 | 0.0139 | 0.0549 | 8.00E-01 | 0.0204 | 0.0635 | 7.48E-01 | -0.0104 | 0.0605 | 9.68E+08 |
|  | G | 0.4872 | 0.0641 | 2.95E-14 | 0.5178 | 0.0616 | 0.00E+00 | 0.5358 | 0.0631 | 0.00E+00 | 0.4919 | 0.0571 | 0.00E+00 | 0.4651 | 0.0566 | 2.22E-16 |
|  | D | 0.0372 | 0.0462 | 4.21E-01 | -0.0463 | 0.0417 | 2.67E-01 | -0.0420 | 0.0474 | 3.76E-01 | -0.0478 | 0.0463 | 3.02E-01 | -0.0410 | 0.0538 | 4.45E-01 |
| H-GCTA | M1 | 0.2576 | 0.0445 | 7.12E-09 | 0.2133 | 0.0457 | 3.03E-06 | 0.2026 | 0.0549 | 2.27E-04 | 0.1640 | 0.0408 | 5.71E-05 | 0.1575 | 0.0435 | 2.98E-04 |
|  | M2 | 0.0138 | 0.0500 | 7.82E-01 | -0.0019 | 0.0358 | 9.57E-01 | -0.0224 | 0.0408 | 5.82E-01 | 0.0182 | 0.0472 | 6.99E-01 | -0.0297 | 0.0443 | 5.03E-01 |
|  | P1 | 0.2416 | 0.0411 | 4.24E-09 | 0.2912 | 0.0399 | 2.91E-13 | 0.3091 | 0.0499 | 6.01E-10 | 0.3462 | 0.0439 | 3.33E-15 | 0.3288 | 0.0456 | 5.86E-13 |
| **B)** |  |  |  |  |  |  |  |  |  |  |  |  |  |  |  |  |
| **25% variants with POEs** |  |  |  |  |  |  |  |  |  |  |  |  |  |  |  |  |
| **Approach** | **GRM** | **m1/p1 = (1.0/1.0)** | | | **m1/p1 = (0.75/1.0)** | | | **m1/p1 = (0.50/1.0)** | | | **m1/p1 = (0.25/1.0)** | | | **m1/p1 = (0.0/1.0)** | | |
|  |  | **ĥ^2^** | **S.E.** | **p-val** | **ĥ^2^** | **S.E.** | **p-val** | **ĥ^2^** | **S.E.** | **p-val** | **ĥ^2^** | **S.E.** | **p-val** | **ĥ^2^** | **S.E.** | **p-val** |
| GCTA | M | 0.1330 | 0.0951 | 1.62E-01 | 0.1600 | 0.0966 | 9.76E-02 | 0.0979 | 0.0860 | 2.55E-01 | 0.0861 | 0.0872 | 3.24E-01 | 0.0352 | 0.0802 | 6.61E-01 |
|  | F | 0.4895 | 0.0951 | 2.66E-07 | 0.4786 | 0.0966 | 7.20E-07 | 0.4944 | 0.0860 | 8.89E-09 | 0.4339 | 0.0872 | 6.47E-07 | 0.4212 | 0.0802 | 1.52E-07 |
| M-GCTA | M' | 0.0085 | 0.0602 | 8.87E-01 | -0.0197 | 0.0573 | 7.31E-01 | -0.0777 | 0.0659 | 2.39E-01 | 0.0282 | 0.0497 | 5.70E-01 | 0.0298 | 0.0665 | 6.55E-01 |
|  | G | 0.5075 | 0.0708 | 7.63E-13 | 0.4716 | 0.0629 | 6.46E-14 | 0.5133 | 0.0541 | 0.00E+00 | 0.4343 | 0.0668 | 7.71E-11 | 0.4835 | 0.0626 | 1.11E-14 |
|  | D | -0.0203 | 0.0467 | 6.64E-01 | 0.0126 | 0.0456 | 7.83E-01 | 0.0017 | 0.0514 | 9.74E-01 | -0.0087 | 0.0492 | 8.60E-01 | -0.0696 | 0.0516 | 1.78E-01 |
| H-GCTA | M1 | 0.2612 | 0.0471 | 2.98E-08 | 0.2338 | 0.0426 | 4.12E-08 | 0.2127 | 0.0455 | 3.00E-06 | 0.2045 | 0.0338 | 1.51E-09 | 0.2033 | 0.0428 | 2.08E-06 |
|  | M2 | -0.0172 | 0.0459 | 7.07E-01 | -0.0002 | 0.0403 | 9.96E-01 | -0.0769 | 0.0414 | 6.32E-02 | 0.0263 | 0.0391 | 5.01E-01 | -0.0158 | 0.0482 | 7.43E-01 |
|  | P1 | 0.2545 | 0.0430 | 3.20E-09 | 0.2685 | 0.0460 | 5.40E-09 | 0.2697 | 0.0414 | 7.12E-11 | 0.2613 | 0.0502 | 1.93E-07 | 0.2782 | 0.0492 | 1.59E-08 |
