# Supplementary material for: Haplotype-based analysis distinguishes maternal-fetal genetic contribution to pregnancy-related outcomes
Source: PLoS Genet. 2025 Mar 10;21(3):e1011575. doi: 10.1371/journal.pgen.1011575 (PMC11918446; doi:10.1371/journal.pgen.1011575)
Supplement: S22 Table — h^2 of simulated traits from pooled dataset with correlated maternal-fetal genetic effects and POEs, estimated through conventional GCTA, M-GCTA and H-GCTA approach using GREML (α = -1.0) model. For simplicity, we assumed that all causal variants exhibit POEs. POEs were incorporated by reducing the effect of m1 as compared to p1 by multiplying effects of m1 with (1 – I) where I is the imprinting factor such as 0.25, 0.50, 0.75 and 1.0. m1 shows either no imprinting, i.e., I = 0.0 (m1/p1=1.0/1.0) or partial imprinting, i.e., I = 0.25-0.75 (m1/p1=0.75/1.0−0.25/1.0) or complete imprinting, i.e., I = 1.0 (m1/p1=0.0/1.0). For GCTA, M is the GRM generated from maternal genotypes (m), and F is the GRM generated from fetal genotypes (f). For M-GCTA, M’ represents the genetic relationship matrix of mothers; G represents genetic relationship matrix of children and D represents mother-child covariance matrix. For H-GCTA, M1 is the GRM generated from maternal transmitted alleles (m1), M2 is the GRM generated from maternal non-transmitted alleles (m2), and P1 is the GRM generated from paternal transmitted alleles (p1). A total of 100 replicates of each phenotype were simulated using empirical genotypes of Pooled dataset. P-values were calculated using z test statistics (two sided). (DOCX) [file pgen.1011575.s023.docx]

# **S22 Table: SNP-based heritability of simulated traits simulated traits from pooled dataset with correlated maternal-fetal genetic effects and parent-of-origin effects (POEs)**

| **Traits with correlated maternal-fetal genetic effects [cor(u_m_, u_f_) = 1.0] and varying levels of maternal imprinting**^†^ | | | | | | | | | | | |  |  |  |  |  |
| --- | --- | --- | --- | --- | --- | --- | --- | --- | --- | --- | --- | --- | --- | --- | --- | --- |
| **Approach** | **GRM** | **u_m1_/u_p1_ = 1.0/1.0** | | | **u_m1_/u_p1_ = 0.75/1.0** | | | **u_m1_/u_p1_ = 0.50/1.0** | | | **u_m1_/u_p1_ = 0.25/1.0** | | | **u_m1_/u_p1_ = 0.0/1.0** | | |
|  |  | **ĥ^2^** | **S.E.** | **p-val** | **ĥ^2^** | **S.E.** | **p-val** | **ĥ^2^** | **S.E.** | **p-val** | **ĥ^2^** | **S.E.** | **p-val** | **ĥ^2^** | **S.E.** | **p-val** |
| GCTA | M | 0.4226 | 0.0727 | 6.13E-09 | 0.5122 | 0.0836 | 8.79E-10 | 0.5158 | 0.0857 | 1.73E-09 | 0.3720 | 0.0906 | 4.04E-05 | 0.2979 | 0.0965 | 2.02E-03 |
|  | F | 0.4362 | 0.0727 | 1.97E-09 | 0.4584 | 0.0836 | 4.12E-08 | 0.4391 | 0.0857 | 2.97E-07 | 0.3247 | 0.0906 | 3.39E-04 | 0.3833 | 0.0965 | 7.12E-05 |
| M-GCTA | M' | 0.2077 | 0.0527 | 8.03E-05 | 0.2966 | 0.0597 | 6.66E-07 | 0.2211 | 0.0554 | 6.65E-05 | 0.1739 | 0.0642 | 6.77E-03 | 0.1442 | 0.0642 | 2.46E-02 |
|  | G | 0.1749 | 0.0513 | 6.43E-04 | 0.2371 | 0.0569 | 3.11E-05 | 0.1544 | 0.0511 | 2.53E-03 | 0.1000 | 0.0601 | 9.61E-02 | 0.2143 | 0.0668 | 1.33E-03 |
|  | D | 0.2079 | 0.0378 | 3.92E-08 | 0.1396 | 0.0394 | 3.96E-04 | 0.2107 | 0.0460 | 4.67E-06 | 0.1849 | 0.0480 | 1.16E-04 | 0.1293 | 0.0478 | 6.82E-03 |
| H-GCTA | M1 | 0.4203 | 0.0417 | 0.00E+00 | 0.3929 | 0.0372 | 0.00E+00 | 0.3465 | 0.0370 | 0.00E+00 | 0.2445 | 0.0481 | 3.65E-07 | 0.1993 | 0.0423 | 2.44E-06 |
|  | M2 | 0.1309 | 0.0443 | 3.13E-03 | 0.1432 | 0.0424 | 7.27E-04 | 0.1497 | 0.0412 | 2.82E-04 | 0.1017 | 0.0434 | 1.91E-02 | 0.1508 | 0.0431 | 4.63E-04 |
|  | P1 | 0.0909 | 0.0372 | 1.47E-02 | 0.1365 | 0.0459 | 2.93E-03 | 0.1098 | 0.0404 | 6.63E-03 | 0.0818 | 0.0389 | 3.58E-02 | 0.2375 | 0.0473 | 4.99E-07 |

† - For simplicity, we assumed that all causal variants exhibit POEs.
