# Supplementary material for: Haplotype-based analysis distinguishes maternal-fetal genetic contribution to pregnancy-related outcomes
Source: PLoS Genet. 2025 Mar 10;21(3):e1011575. doi: 10.1371/journal.pgen.1011575 (PMC11918446; doi:10.1371/journal.pgen.1011575)
Supplement: S24 Table — Comparison of h^2 estimated through conventional GCTA, M-GCTA and H-GCTA approach for A) gestational duration, B) birth weight, C) birth length and D) head circumference. Each approach was fitted using GREML (α = -0.25, -1.0), LDAK-Thin (α = -0.25, -1.0) and LDAK-Weights (α = -0.25, -1.0). For GCTA, M is the GRM generated from maternal genotypes (m), and F is the GRM generated from fetal genotypes (f). For M-GCTA, M’ represents the genetic relationship matrix of mothers; G represents genetic relationship matrix of children and D represents mother-child covariance matrix. For H-GCTA, M1 is the GRM generated from maternal transmitted alleles (m1), M2 is the GRM generated from maternal non-transmitted alleles (m2), and P1 is the GRM generated from paternal transmitted alleles (p1). Gestational duration was adjusted for fetal sex and fetal size measurements at birth were additionally adjusted for gestational duration up to third orthogonal polynomial. Analyses using GCTA and M-GCTA approach were adjusted for 20 PCs and H-GCTA approach was adjusted for 30 PCs (10 PCs corresponding to m1, m2 and p1 each). P-values were calculated using z test statistics (one sided). (DOCX) [file pgen.1011575.s025.docx]

# **S24 Table: SNP-based heritability of gestational duration and fetal size measurements at birth using SNPs with MAF > 0.001**

**A)**

| **h^2^ for gestational duration** |  |  |  |  |  |  |  |  |  |  |  |  |  |  |  |  |  |  |  |  |  |
| --- | --- | --- | --- | --- | --- | --- | --- | --- | --- | --- | --- | --- | --- | --- | --- | --- | --- | --- | --- | --- | --- |
| MAF Cut-off | Approach | GRM | GREML (alpha = -1.0) | | | | GREML (alpha = -0.25) | | | LDAK-Thin (alpha = -1.0) | | | LDAK-Thin (alpha = -0.25) | | | LDAK-Weights (alpha = -1.0) | | | LDAK-Weights (alpha = -0.25) | | |
|  |  |  | ĥ^2^ | | S.E. | p-val | ĥ^2^ | SD | p-val | ĥ^2^ | SD | p-val | ĥ^2^ | SD | p-val | ĥ^2^ | SD | p-val | ĥ^2^ | SD | p-val |
| MAF > 0.001 | GCTA | M | | 0.2556 | 0.0475 | 3.78E-08 | 0.1708 | 0.0349 | 5.10E-07 | 0.4321 | 0.0723 | 1.14E-09 | 0.2598 | 0.0469 | 1.48E-08 | 0.3468 | 0.0921 | 8.39E-05 | 0.3898 | 0.0823 | 1.09E-06 |
|  |  | F | | 0.0976 | 0.0455 | 1.60E-02 | 0.0497 | 0.0328 | 6.51E-02 | 0.1871 | 0.0720 | 4.67E-03 | 0.1021 | 0.0461 | 1.33E-02 | 0.1572 | 0.0925 | 4.46E-02 | 0.2289 | 0.0822 | 2.68E-03 |
|  | M-GCTA | M' | | 0.2362 | 0.0616 | 6.37E-05 | 0.1751 | 0.0454 | 5.70E-05 | 0.3706 | 0.0948 | 4.63E-05 | 0.2590 | 0.0609 | 1.07E-05 | 0.2068 | 0.1251 | 4.91E-02 | 0.3535 | 0.0859 | 1.94E-05 |
|  |  | G | | 0.0356 | 0.0587 | 2.72E-01 | 0.0217 | 0.0424 | 3.04E-01 | 0.0516 | 0.0938 | 2.91E-01 | 0.0580 | 0.0594 | 1.65E-01 | 0.0075 | 0.1262 | 4.76E-01 | 0.1119 | 0.0850 | 9.42E-02 |
|  |  | D | | 0.0085 | 0.0480 | 4.30E-01 | 0.0001 | 0.0350 | 4.99E-01 | 0.0087 | 0.0760 | 4.54E-01 | 0.0097 | 0.0475 | 4.19E-01 | 0.0348 | 0.1053 | 3.71E-01 | 0.0000 | 0.0000 | NA |
|  | H-GCTA | M1 | | 0.1412 | 0.0458 | 1.03E-03 | 0.1027 | 0.0332 | 9.78E-04 | 0.2055 | 0.0717 | 2.09E-03 | 0.1371 | 0.0453 | 1.24E-03 | 0.0541 | 0.0942 | 2.83E-01 | 0.1090 | 0.0815 | 9.06E-02 |
|  |  | M2 | | 0.1054 | 0.0457 | 1.06E-02 | 0.0732 | 0.0332 | 1.36E-02 | 0.1810 | 0.0719 | 5.92E-03 | 0.1405 | 0.0459 | 1.11E-03 | 0.0368 | 0.0934 | 3.47E-01 | 0.1223 | 0.0824 | 6.89E-02 |
|  |  | P1 | | 0.0234 | 0.0443 | 2.99E-01 | 0.0105 | 0.0317 | 3.71E-01 | 0.0772 | 0.0717 | 1.41E-01 | 0.0514 | 0.0453 | 1.28E-01 | 0.0830 | 0.0944 | 1.90E-01 | 0.1575 | 0.0823 | 2.79E-02 |

**B)**

| **h^2^ for birth weight** |  |  |  |  |  |  |  |  |  |  |  |  |  |  |  |  |  |  |  |  |
| --- | --- | --- | --- | --- | --- | --- | --- | --- | --- | --- | --- | --- | --- | --- | --- | --- | --- | --- | --- | --- |
| MAF Cut-off | Approach | GRM | GREML (alpha = -1.0) | | | GREML (alpha = -0.25) | | | LDAK-Thin (alpha = -1.0) | | | LDAK-Thin (alpha = -0.25) | | | LDAK-Weights (alpha = -1.0) | | | LDAK-Weights (alpha = -0.25) | | |
|  |  |  | ĥ^2^ | S.E. | p-val | ĥ^2^ | SD | p-val | ĥ^2^ | SD | p-val | ĥ^2^ | SD | p-val | ĥ^2^ | SD | p-val | ĥ^2^ | SD | p-val |
| MAF > 0.001 | GCTA | M | 0.1634 | 0.0534 | 1.10E-03 | 0.1241 | 0.0385 | 6.34E-04 | 0.2169 | 0.0825 | 4.27E-03 | 0.1475 | 0.0525 | 2.47E-03 | 0.2687 | 0.1038 | 4.80E-03 | 0.2599 | 0.0928 | 2.55E-03 |
|  |  | F | 0.3246 | 0.0543 | 1.13E-09 | 0.2242 | 0.0396 | 7.46E-09 | 0.4184 | 0.0821 | 1.71E-07 | 0.2716 | 0.0529 | 1.40E-07 | 0.3098 | 0.1029 | 1.30E-03 | 0.3740 | 0.0937 | 3.26E-05 |
|  | M-GCTA | M' | 0.0564 | 0.0665 | 1.98E-01 | 0.0590 | 0.0485 | 1.12E-01 | 0.0606 | 0.1059 | 2.84E-01 | 0.0694 | 0.0676 | 1.52E-01 | 0.1690 | 0.1405 | 1.15E-01 | 0.1493 | 0.1230 | 1.12E-01 |
|  |  | G | 0.2709 | 0.0693 | 4.69E-05 | 0.1952 | 0.0505 | 5.61E-05 | 0.3497 | 0.1078 | 5.89E-04 | 0.2417 | 0.0690 | 2.31E-04 | 0.2358 | 0.1406 | 4.67E-02 | 0.3137 | 0.1237 | 5.59E-03 |
|  |  | D | 0.0424 | 0.0529 | 2.12E-01 | 0.0141 | 0.0387 | 3.58E-01 | 0.0563 | 0.0845 | 2.53E-01 | 0.0138 | 0.0533 | 3.98E-01 | 0.0068 | 0.1166 | 4.77E-01 | 0.0133 | 0.0987 | 4.46E-01 |
|  | H-GCTA | M1 | 0.1919 | 0.0533 | 1.60E-04 | 0.1286 | 0.0385 | 4.26E-04 | 0.2581 | 0.0818 | 7.97E-04 | 0.1660 | 0.0525 | 7.83E-04 | 0.1623 | 0.1052 | 6.14E-02 | 0.2921 | 0.0935 | 8.91E-04 |
|  |  | M2 | 0.0335 | 0.0495 | 2.49E-01 | 0.0405 | 0.0357 | 1.29E-01 | 0.0000 | 0.0000 | NA | 0.0276 | 0.0498 | 2.89E-01 | 0.0021 | 0.1042 | 4.92E-01 | 0.0214 | 0.0907 | 4.07E-01 |
|  |  | P1 | 0.1360 | 0.0520 | 4.45E-03 | 0.0859 | 0.0375 | 1.09E-02 | 0.1680 | 0.0810 | 1.90E-02 | 0.1053 | 0.0512 | 1.99E-02 | 0.1222 | 0.1051 | 1.23E-01 | 0.1126 | 0.0921 | 1.11E-01 |

**C)**

| **h^2^ for birth length** |  |  |  |  |  |  |  |  |  |  |  |  |  |  |  |  |  |  |  |  |  |
| --- | --- | --- | --- | --- | --- | --- | --- | --- | --- | --- | --- | --- | --- | --- | --- | --- | --- | --- | --- | --- | --- |
| MAF Cut-off | Approach | GRM | GREML (alpha = -1.0) | | | | GREML (alpha = -0.25) | | | LDAK-Thin (alpha = -1.0) | | | LDAK-Thin (alpha = -0.25) | | | LDAK-Weights (alpha = -1.0) | | | LDAK-Weights (alpha = -0.25) | | |
|  |  |  | ĥ^2^ | | S.E. | p-val | ĥ^2^ | SD | p-val | ĥ^2^ | SD | p-val | ĥ^2^ | SD | p-val | ĥ^2^ | SD | p-val | ĥ^2^ | SD | p-val |
| MAF > 0.001 | GCTA | M | | 0.2254 | 0.0741 | 1.18E-03 | 0.1503 | 0.0535 | 2.49E-03 | 0.1978 | 0.1137 | 4.10E-02 | 0.1272 | 0.0732 | 4.11E-02 | 0.1814 | 0.1366 | 9.22E-02 | 0.2046 | 0.1286 | 5.59E-02 |
|  |  | F | | 0.2664 | 0.0749 | 1.89E-04 | 0.1589 | 0.0543 | 1.70E-03 | 0.4306 | 0.1154 | 9.54E-05 | 0.2370 | 0.0745 | 7.34E-04 | 0.4850 | 0.1390 | 2.43E-04 | 0.4625 | 0.1306 | 1.98E-04 |
|  | M-GCTA | M' | | 0.0026 | 0.0929 | 4.89E-01 | 0.0115 | 0.0664 | 4.31E-01 | 0.0095 | 0.1497 | 4.75E-01 | 0.0093 | 0.0956 | 4.61E-01 | 0.0092 | 0.1905 | 4.81E-01 | 0.0075 | 0.1733 | 4.83E-01 |
|  |  | G | | 0.0452 | 0.0938 | 3.15E-01 | 0.0211 | 0.0669 | 3.76E-01 | 0.0137 | 0.1513 | 4.64E-01 | 0.0117 | 0.0958 | 4.52E-01 | 0.1129 | 0.1958 | 2.82E-01 | 0.0589 | 0.1764 | 3.69E-01 |
|  |  | D | | 0.2078 | 0.0717 | 1.89E-03 | 0.1294 | 0.0513 | 5.80E-03 | 0.2310 | 0.1206 | 2.77E-02 | 0.1494 | 0.0750 | 2.32E-02 | 0.0813 | 0.1673 | 3.14E-01 | 0.1525 | 0.1415 | 1.41E-01 |
|  | H-GCTA | M1 | | 0.2425 | 0.0740 | 5.26E-04 | 0.1407 | 0.0530 | 3.97E-03 | 0.2128 | 0.1137 | 3.06E-02 | 0.0997 | 0.0723 | 8.39E-02 | 0.2825 | 0.1457 | 2.63E-02 | 0.2824 | 0.1301 | 1.50E-02 |
|  |  | M2 | | 0.0093 | 0.0710 | 4.48E-01 | 0.0042 | 0.0511 | 4.67E-01 | 0.0089 | 0.1124 | 4.68E-01 | 0.0074 | 0.0729 | 4.59E-01 | 0.0097 | 0.1431 | 4.73E-01 | 0.0090 | 0.1303 | 4.73E-01 |
|  |  | P1 | | 0.0464 | 0.0716 | 2.59E-01 | 0.0297 | 0.0522 | 2.84E-01 | 0.0210 | 0.1137 | 4.27E-01 | 0.0331 | 0.0727 | 3.25E-01 | 0.0127 | 0.1468 | 4.65E-01 | 0.0212 | 0.1316 | 4.36E-01 |

**D)**

| **h^2^ for head circumference** |  |  |  |  |  |  |  |  |  |  |  |  |  |  |  |  |  |  |  |  |  |
| --- | --- | --- | --- | --- | --- | --- | --- | --- | --- | --- | --- | --- | --- | --- | --- | --- | --- | --- | --- | --- | --- |
| MAF Cut-off | Approach | GRM | GREML (alpha = -1.0) | | | | GREML (alpha = -0.25) | | | LDAK-Thin (alpha = -1.0) | | | LDAK-Thin (alpha = -0.25) | | | LDAK-Weights (alpha = -1.0) | | | LDAK-Weights (alpha = -0.25) | | |
|  |  |  | ĥ^2^ | | S.E. | p-val | ĥ^2^ | SD | p-val | ĥ^2^ | SD | p-val | ĥ^2^ | SD | p-val | ĥ^2^ | SD | p-val | ĥ^2^ | SD | p-val |
| MAF > 0.001 | GCTA | M | | 0.3238 | 0.0906 | 1.76E-04 | 0.2276 | 0.0654 | 2.51E-04 | 0.4090 | 0.1408 | 1.84E-03 | 0.2497 | 0.0891 | 2.54E-03 | 0.3581 | 0.1899 | 2.97E-02 | 0.3530 | 0.1590 | 1.32E-02 |
|  |  | F | | 0.4049 | 0.0924 | 5.93E-06 | 0.2595 | 0.0663 | 4.51E-05 | 0.6205 | 0.1434 | 7.61E-06 | 0.3114 | 0.0909 | 3.08E-04 | 0.6276 | 0.1799 | 2.43E-04 | 0.5906 | 0.1612 | 1.24E-04 |
|  | M-GCTA | M' | | 0.0791 | 0.1116 | 2.39E-01 | 0.0432 | 0.0780 | 2.90E-01 | 0.0221 | 0.1819 | 4.52E-01 | 0.0022 | 0.1128 | 4.92E-01 | 0.0011 | 0.2586 | 4.98E-01 | 0.0080 | 0.2144 | 4.85E-01 |
|  |  | G | | 0.1850 | 0.1158 | 5.51E-02 | 0.0796 | 0.0823 | 1.67E-01 | 0.3348 | 0.1862 | 3.61E-02 | 0.0921 | 0.1168 | 2.15E-01 | 0.4621 | 0.2432 | 2.87E-02 | 0.4260 | 0.2139 | 2.32E-02 |
|  |  | D | | 0.2118 | 0.0877 | 7.86E-03 | 0.1737 | 0.0611 | 2.22E-03 | 0.3032 | 0.1457 | 1.87E-02 | 0.2346 | 0.0896 | 4.41E-03 | 0.1198 | 0.2068 | 2.81E-01 | 0.1560 | 0.1735 | 1.84E-01 |
|  | H-GCTA | M1 | | 0.3277 | 0.0916 | 1.73E-04 | 0.2020 | 0.0658 | 1.07E-03 | 0.4378 | 0.1423 | 1.05E-03 | 0.2085 | 0.0900 | 1.03E-02 | 0.2287 | 0.1864 | 1.10E-01 | 0.3664 | 0.1599 | 1.10E-02 |
|  |  | M2 | | 0.0682 | 0.0875 | 2.18E-01 | 0.0371 | 0.0620 | 2.75E-01 | 0.0138 | 0.1386 | 4.60E-01 | 0.0386 | 0.0865 | 3.28E-01 | 0.0096 | 0.1996 | 4.81E-01 | 0.0779 | 0.1624 | 3.16E-01 |
|  |  | P1 | | 0.1280 | 0.0885 | 7.41E-02 | 0.0576 | 0.0631 | 1.81E-01 | 0.1673 | 0.1392 | 1.15E-01 | 0.0420 | 0.0877 | 3.16E-01 | 0.0190 | 0.1768 | 4.57E-01 | 0.1309 | 0.1582 | 2.04E-01 |
