# Supplementary material for: Haplotype-based analysis distinguishes maternal-fetal genetic contribution to pregnancy-related outcomes
Source: PLoS Genet. 2025 Mar 10;21(3):e1011575. doi: 10.1371/journal.pgen.1011575 (PMC11918446; doi:10.1371/journal.pgen.1011575)
Supplement: S27 Table — h^2 of gestational duration in HARVEST dataset based on SNPs with MAF > 0.01estimated through H-GCTA using GREML (α = -1.0) model. Gestational duration was adjusted for fetal sex. P-values were calculated using z test statistics (one sided). (DOCX) [file pgen.1011575.s028.docx]

# **S27 Table: Replication of heritability estimation of gestational duration**

| **h^2^ for gestational duration (days) in HARVEST/MoBa (7.5 million SNPs)** |  |  |  |  |  |
| --- | --- | --- | --- | --- | --- |
| MAF Cut-off | Approach | GRM | ĥ^2^ | S.E. | p-val |
| MAF > 0.01 | H-GCTA | M1 | 0.0768 | 0.0382 | 2.22E-02 |
|  |  | M2 | 0.0431 | 0.0382 | 1.30E-01 |
|  |  | P1 | 0.0249 | 0.0368 | 2.49E-01 |
