# Supplementary material for: Haplotype-based analysis distinguishes maternal-fetal genetic contribution to pregnancy-related outcomes
Source: PLoS Genet. 2025 Mar 10;21(3):e1011575. doi: 10.1371/journal.pgen.1011575 (PMC11918446; doi:10.1371/journal.pgen.1011575)
Supplement: S1 Fig — Framework of the study depicting the traits under study, available datasets, MAF cutoffs, list of GRMs created in each MAF cutoff category, selection of unrelated mother-child pairs. Last block shows methods/models utilized for estimation and comparison of h^2 estimated from our approach (H-GCTA) with those obtained by two available approaches – GCTA and M-GCTA. (PDF) [file pgen.1011575.s029.pdf]

S1 Fig: Framework of the study

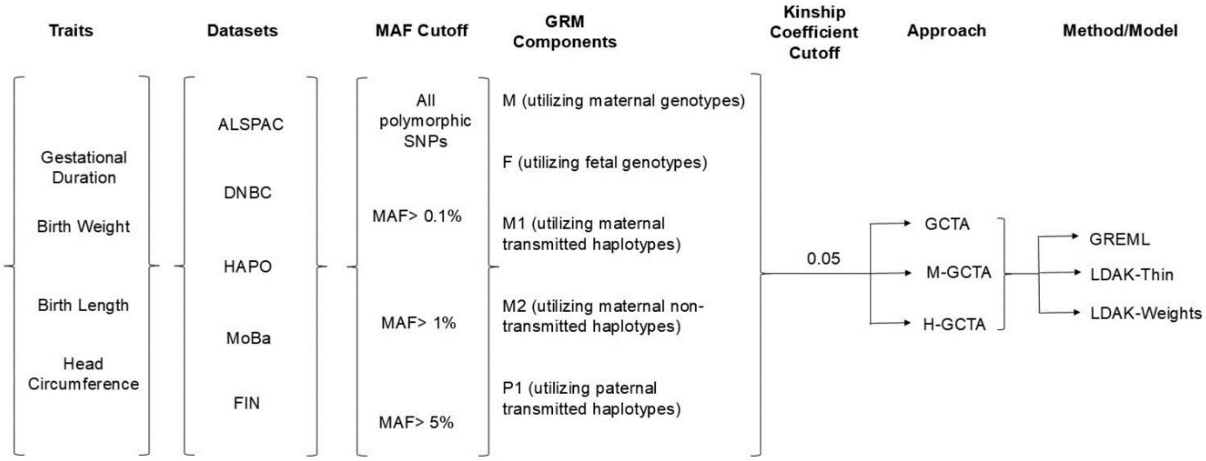

Framework of the study depicting the traits under study, available datasets, MAF cutoffs, list of GRMs created in each MAF cutoff category, selection of unrelated mother-child pairs. Last block shows methods/models utilized for estimation and comparison of $\hat{h}^2$ estimated from our approach (H-GCTA) with those obtained by two available approaches – GCTA and M-GCTA.
