# Supplementary material for: Haplotype-based analysis distinguishes maternal-fetal genetic contribution to pregnancy-related outcomes
Source: PLoS Genet. 2025 Mar 10;21(3):e1011575. doi: 10.1371/journal.pgen.1011575 (PMC11918446; doi:10.1371/journal.pgen.1011575)
Supplement: S2 Fig — Distribution of available phenotypes in each dataset categorized by fetal sex – A) distribution of gestational duration, birth weight, birth length and head circumference in ALSPAC dataset; B) distribution of gestational duration, birth weight, birth length and head circumference in HAPO dataset; C) distribution of gestational duration, birth weight and birth length in FIN dataset; D) distribution of gestational duration and birth weight in DNBC dataset and E) distribution of gestational duration in MoBa dataset. (PDF) [file pgen.1011575.s030.pdf]

S2 Fig: Distribution of available phenotypes in datasets

A) Phenotypes distribution in ALSPAC dataset

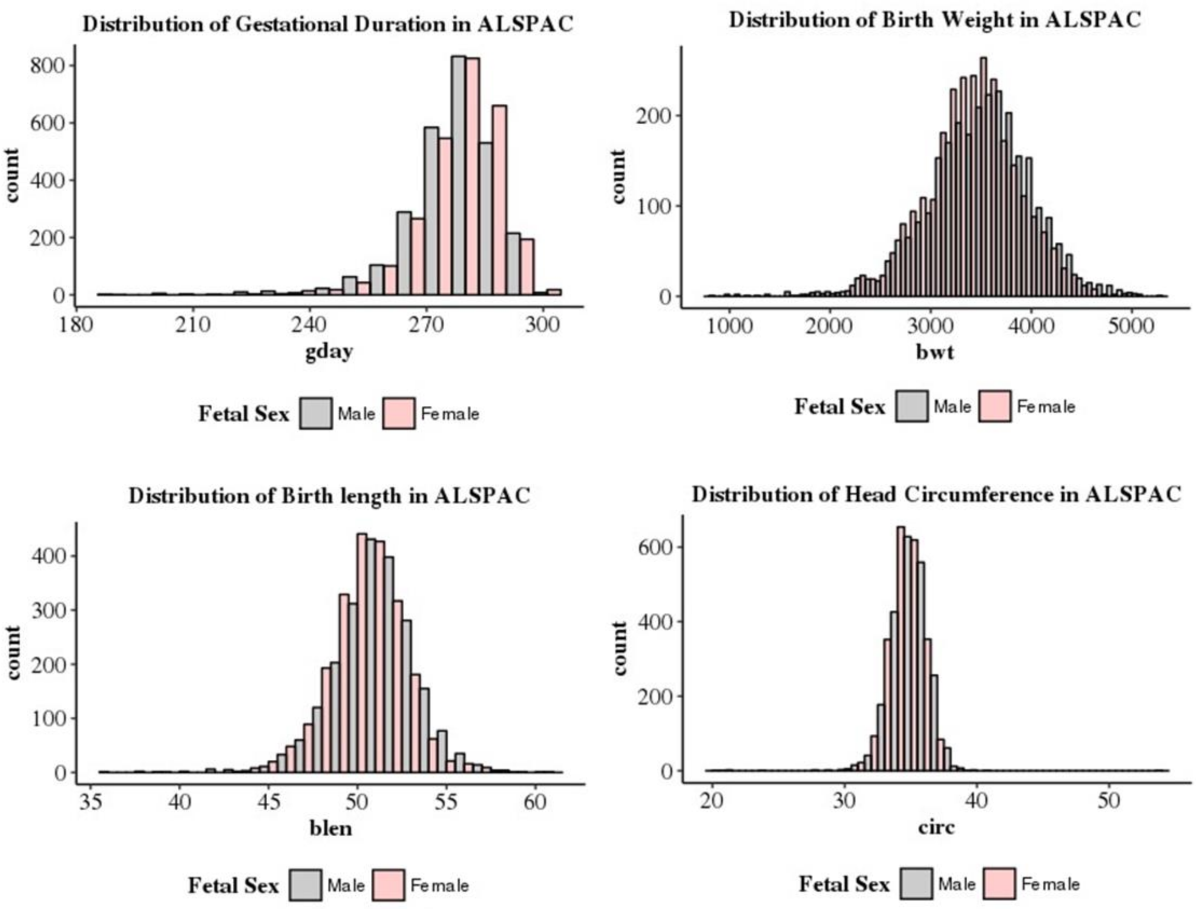

B) Phenotypes distribution in HAPO dataset

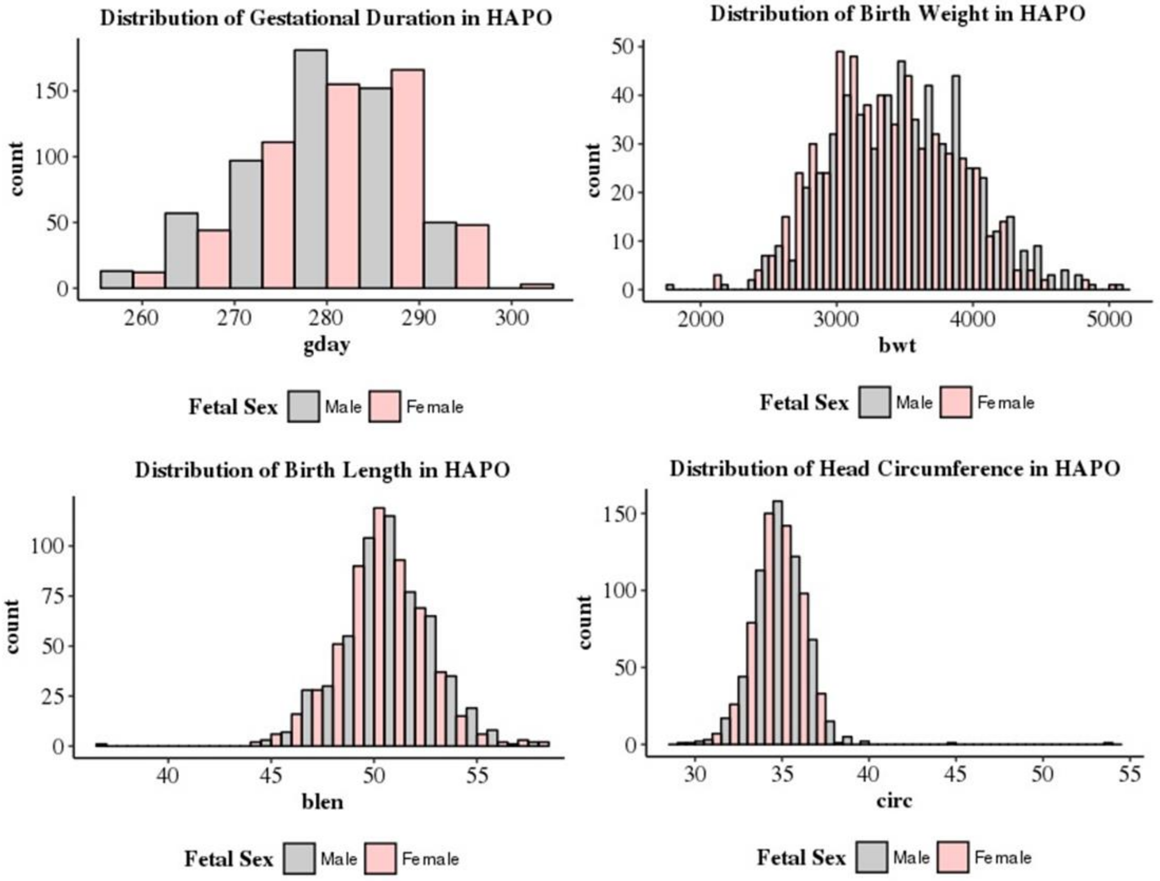

C) Phenotypes distribution in FIN dataset

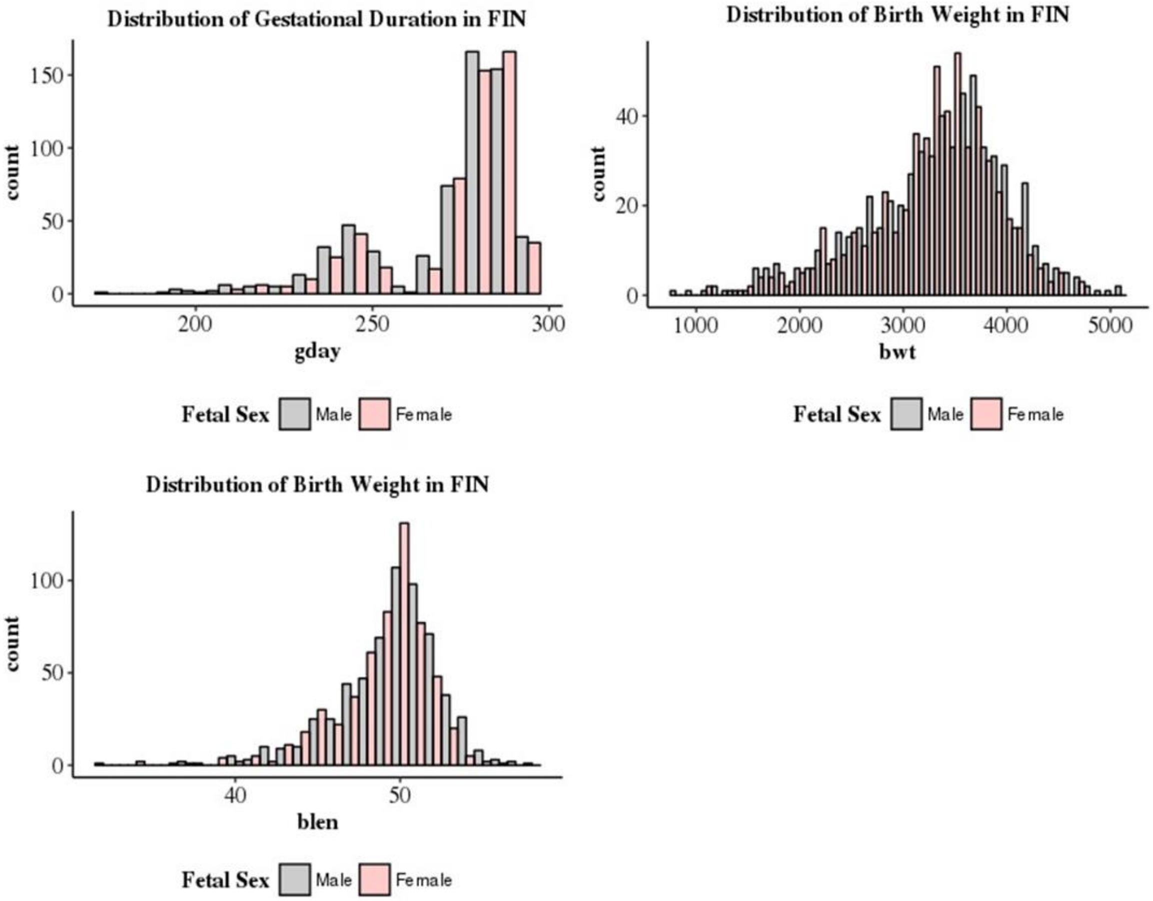

D) Phenotypes distribution in DNBC dataset

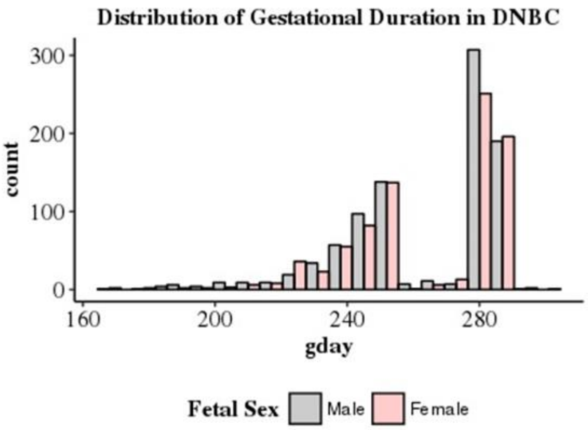

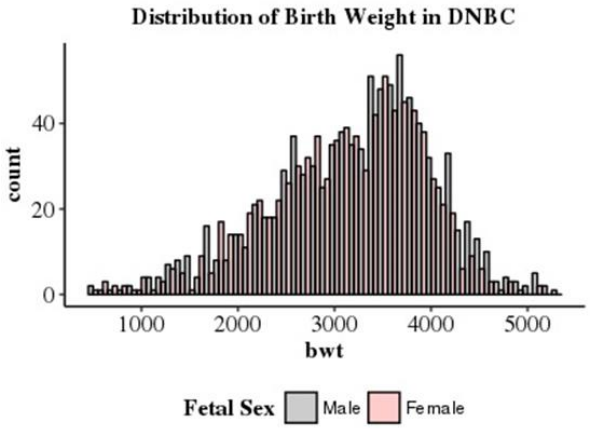

E) Phenotypes distribution in MoBa dataset

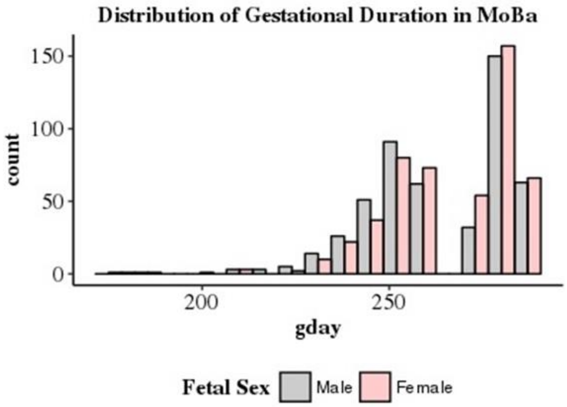

Distribution of available phenotypes in each dataset categorized by fetal sex – A) distribution of gestational duration, birth weight, birth length and head circumference in ALSPAC dataset; B) distribution of gestational duration, birth weight, birth length and head circumference in HAPO dataset; C) distribution of gestational duration, birth weight and birth length in FIN dataset; D) distribution of gestational duration and birth weight in DNBC dataset and E) distribution of gestational duration in MoBa dataset.
