# Supplementary material for: Haplotype-based analysis distinguishes maternal-fetal genetic contribution to pregnancy-related outcomes
Source: PLoS Genet. 2025 Mar 10;21(3):e1011575. doi: 10.1371/journal.pgen.1011575 (PMC11918446; doi:10.1371/journal.pgen.1011575)
Supplement: S3 Fig — Comparison of h^2 for simulated traits from ALSPAC dataset, estimated through different approaches fitting GREML (α = -1.0): A) maternal traits; B) fetal traits; C) traits where independent sets of causal variants have effects through mother and fetus; D) traits where same set of causal variants have effects through mother and fetus. For GCTA, M is the GRM generated from maternal genotypes (m), and F is the GRM generated from fetal genotypes (f). For M-GCTA, M’ represents the genetic relationship matrix of mothers; G represents genetic relationship matrix of children and D represents mother-child covariance matrix. For H-GCTA, M1 is the GRM generated from maternal transmitted alleles (m1), M2 is the GRM generated from maternal non-transmitted alleles (m2), and P1 is the GRM generated from paternal transmitted alleles (p1). A total of 100 replicates of each phenotype were simulated using empirical genotypes of ALSPAC dataset. P-values were calculated using z test statistics (two sided). * = (p value <5.0E-02), ** = (p value <1.0E-02), *** = (p value <1.0E-03) and **** = (p value <1.0E-04). (PDF) [file pgen.1011575.s031.pdf]

S3 Fig: Comparison of $\hat{h}^2$ for simulated traits from ALSPAC dataset – maternal traits, fetal traits and traits with independent maternal-fetal genetic effects

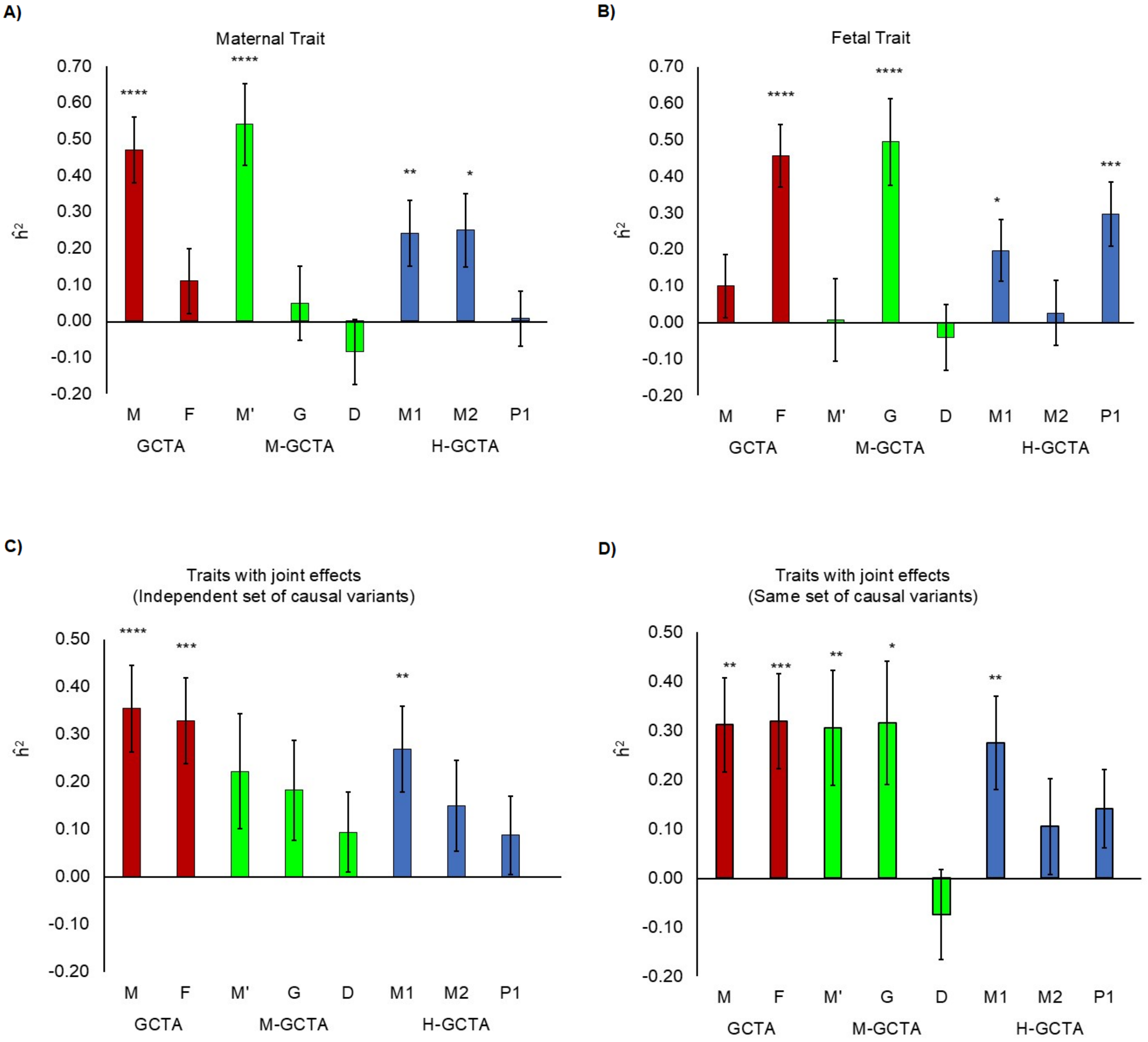

Comparison of $\hat{h}^2$ for simulated traits from ALSPAC dataset, estimated through different approaches fitting GREML (α = -1.0): A) maternal traits; B) fetal traits; C) traits where independent sets of causal variants have effects through mother and fetus; D) traits where same set of causal variants have effects through mother and fetus. For GCTA, M is the GRM generated from maternal genotypes (m), and F is the GRM generated from fetal genotypes (f). For M-GCTA, M' represents the genetic relationship matrix of mothers; G represents genetic relationship matrix of children and D represents mother-child covariance matrix. For H-GCTA, M1 is the GRM generated from maternal transmitted alleles (m1), M2 is the GRM generated from maternal non-transmitted alleles (m2), and P1 is the GRM generated from paternal transmitted alleles (p1). A total of 100 replicates of each phenotype were simulated using empirical genotypes of ALSPAC dataset. P-values were calculated using z test statistics (two sided). * = (p value < 5.0E-02), ** = (p value < 1.0E-02), *** = (p value < 1.0E-03) and **** = (p value < 1.0E-04).
