# Supplementary material for: Haplotype-based analysis distinguishes maternal-fetal genetic contribution to pregnancy-related outcomes
Source: PLoS Genet. 2025 Mar 10;21(3):e1011575. doi: 10.1371/journal.pgen.1011575 (PMC11918446; doi:10.1371/journal.pgen.1011575)
Supplement: S5 Fig — Schematic representation of variance attributable to maternal transmitted (σm12), maternal non-transmitted (σm22) and paternal transmitted (σp12) haplotypes in H-GCTA – Z and W are the sets of causal variants with maternal and fetal effects, respectively. S1 (Black squares), S2 (orange circles) and S3 (purple triangles) are sets of causal variants with explicit maternal effects, joint-maternal-fetal effects and explicit fetal effects such that S1 ∈ Z, S2 = Z ∩ W and S3 ∈ W. um and uf are causal effects through mother and fetus and pm and pf are reference allele frequencies of a causal variant in mother and fetus, respectively. Since each allele is a random draw from Bernoulli distribution, variance in terms of allele frequency is represented as pm1−pm and pf1−pf in mother and fetus, respectively. m1 affects the phenotype through maternal transmitted alleles in mother (gm1') and maternal transmitted alleles in fetus (gm1''). Likewise, gm2 and gp1 represent the maternal non-transmitted and paternal transmitted alleles. Therefore, allelic effects - um1'=um2, um1''=up1 (in the absence of POEs) and covgm1',gm1''=ρgm1',gm1''σgm1'σgm1'' is the covariance of two binomial random variables m1’ and m1” present in mother and fetus, respectively; where, ρ and σ represent correlation and standard deviation of respective alleles. For a causal variant with joint maternal-fetal effect, pm=pf=p in a random mating population, therefore, covgm1',gm1''=p1−p and total phenotypic variance explained by m1, m2 and p1 is 2p1−pum2+uf2+umuf. (PDF) [file pgen.1011575.s033.pdf]

S5 Fig: Schematic representation of variance attributable to maternal transmitted, maternal non-transmitted and paternal transmitted haplotypes in H-GCTA

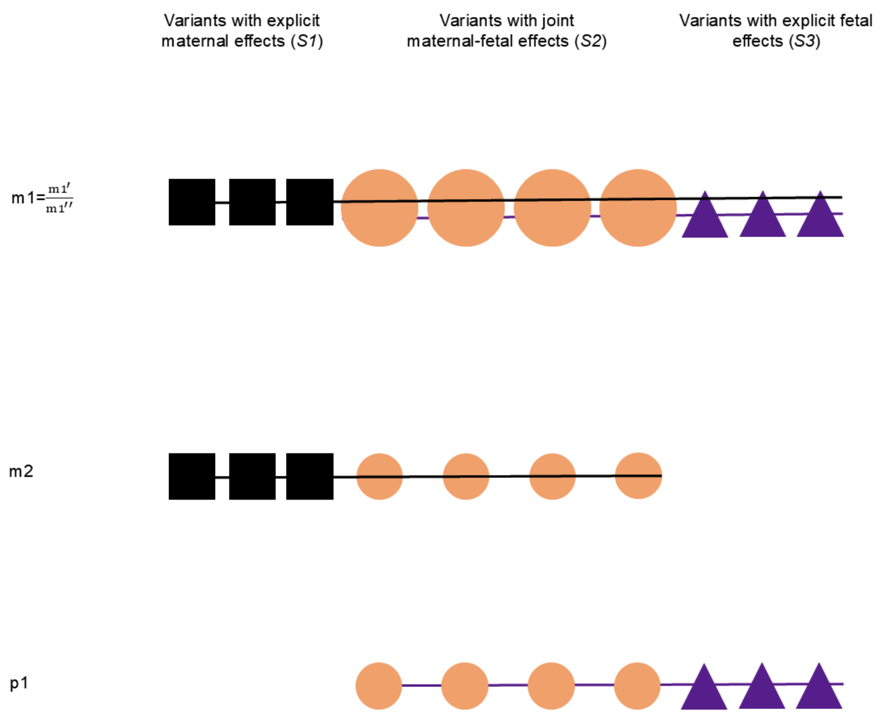

$$\sigma_{m1}^2 = \sum_{S1 \in Z} var(u_m g_{m1'}) + \sum_{S2 = Z \cap W} var(u_m g_{m1'} + u_f g_{m1''}) + \sum_{S3 \in W} var(u_f g_{m1''})$$
$$= \sum_{S1 \in Z} p_m(1 - p_m)(u_m)^2$$
$$+ \sum_{S2 = Z \cap W} p_m(1 - p_m)(u_m)^2 + \sum_{S2 = Z \cap W} 2u_m u_f cov(g_{m1'}, g_{m1''}) + \sum_{S2 = Z \cap W} p_f(1 - p_f)(u_f)^2$$
$$+ \sum_{S3 \in W} p_f(1 - p_f)(u_f)^2$$

$$\sigma_{m2}^2 = \sum_{Z} var(u_m g_{m2}) = \sum_{Z} p_m(1 - p_m)(u_m)^2$$
$$= \sum_{S1 \in Z} p_m(1 - p_m)(u_m)^2$$
$$+ \sum_{S2 = Z \cap W} p_m(1 - p_m)(u_m)^2$$

$$\sigma_{p1}^2 = \sum_{W} var(u_f g_{p1}) = \sum_{W} p_f(1 - p_f)(u_f)^2$$
$$= \sum_{S2 = Z \cap W} p_f(1 - p_f)(u_f)^2$$
$$+ \sum_{S3 \in W} p_f(1 - p_f)(u_f)^2$$

Schematic representation of variance attributable to maternal transmitted (σ^2_{m1}), maternal non-transmitted (σ^2_{m2}) and paternal transmitted (σ^2_{p1}) haplotypes in H-GCTA – Z and W are the sets of causal variants with maternal and fetal effects, respectively. S1 (Black squares), S2 (orange circles) and S3 (purple triangles) are sets of causal variants with explicit maternal effects, joint-maternal-fetal effects and explicit fetal effects such that $S1 \in Z$, $S2 = Z \cap W$ and $S3 \in W$. u_m and u_f are causal effects through mother and fetus and p_m and p_f are reference allele frequencies of a causal variant in mother and fetus, respectively. Since each allele is a random draw from Bernoulli distribution, variance in terms of allele frequency is represented as $p_m(1 - p_m)$ and $p_f(1 - p_f)$ in mother and fetus, respectively. m1 affects the phenotype through maternal transmitted alleles in mother ($g_{m1'}$) and maternal transmitted alleles in fetus ($g_{m1''}$). Likewise, g_{m2} and g_{p1} represent the maternal non-transmitted and paternal transmitted alleles. Therefore, allelic effects - $u_{m1'} = u_{m2}$, $u_{m1''} = u_{p1}$ (in the absence of POEs) and $cov(g_{m1'}, g_{m1''}) = \rho(g_{m1'}, g_{m1''})\sigma_{g_{m1'}}\sigma_{g_{m1''}}$ is the covariance of two binomial random variables m1' and m1'' present in mother and fetus, respectively; where, ρ and σ represent correlation and standard deviation of respective alleles. For a causal variant with joint maternal-fetal effect, $p_m = p_f = p$ in a random mating population, therefore, $cov(g_{m1'}, g_{m1''}) = p(1 - p)$ and total phenotypic variance explained by m1, m2 and p1 is $2p(1 - p)(u_m^2 + u_f^2 + u_m u_f)$.
