# Supplementary material for: Haplotype-based analysis distinguishes maternal-fetal genetic contribution to pregnancy-related outcomes
Source: PLoS Genet. 2025 Mar 10;21(3):e1011575. doi: 10.1371/journal.pgen.1011575 (PMC11918446; doi:10.1371/journal.pgen.1011575)
Supplement: S6 Fig — Principal Components Analysis (PCA) plots of unrelated (relatedness coefficient < 0.5) mother-child pairs using all polymorphic SNPs. m: Mothers’ Genotypes; f: children’s genotypes; m1: maternal transmitted alleles; m2: maternal non-transmitted alleles; and p1: paternal transmitted alleles. Unlike PCA using pooled data, 20 PCs were created using independent SNPs from a merged dataset which included SNPs from 1000 genome samples (phase 3) and pooled dataset. Since 1000 genome dataset in general lacks parent-child information, we used the first allele of phased 1000 genome data along with m1 or p1 to create 20 PCs whereas second allele of phased 1000 genome data was used along with m2 to create 20 PCs. (PDF) [file pgen.1011575.s034.pdf]

S6 Fig: Principal Components Analysis (PCA) plots using all polymorphic SNPs

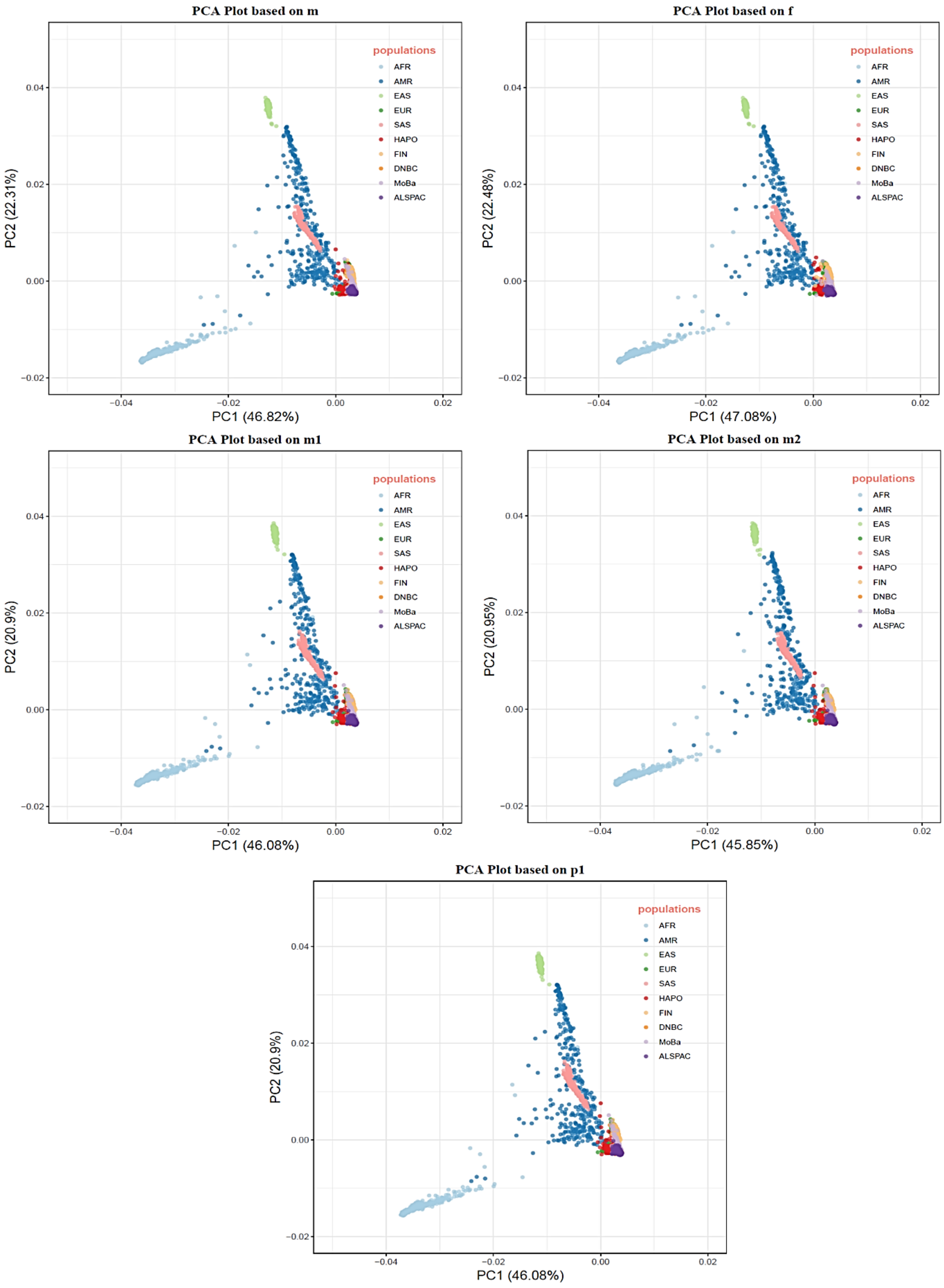

Principal Components Analysis (PCA) plots of unrelated (relatedness coefficient < 0.5) mother-child pairs using all polymorphic SNPs. m: Mothers' Genotypes; f: children's genotypes; m1: maternal transmitted alleles; m2: maternal non-transmitted alleles; and p1: paternal transmitted alleles. Unlike PCA using pooled data, 20 PCs were created using independent SNPs from a merged dataset which included SNPs from 1000 genome samples (phase 3) and pooled dataset. Since 1000 genome dataset in general lacks parent-child information, we used the first allele of phased 1000 genome data along with m1 or p1 to create 20 PCs whereas second allele of phased 1000 genome data was used along with m2 to create 20 PCs.
