# Supplementary material for: Haplotype-based analysis distinguishes maternal-fetal genetic contribution to pregnancy-related outcomes
Source: PLoS Genet. 2025 Mar 10;21(3):e1011575. doi: 10.1371/journal.pgen.1011575 (PMC11918446; doi:10.1371/journal.pgen.1011575)
Supplement: S7 Fig — h^2 estimation of fetal sex adjusted gestational duration in HARVEST dataset using our approach (H-GCTA). Estimated h^2 of fetal sex adjusted gestational duration in pooled dataset is pasted for comparison (image on the right). Analysis was performed through GREML (α = -1.0) using SNPs with MAF > 0.01. (PDF) [file pgen.1011575.s035.pdf]

S7 Fig: Replication of heritability estimation of gestational duration

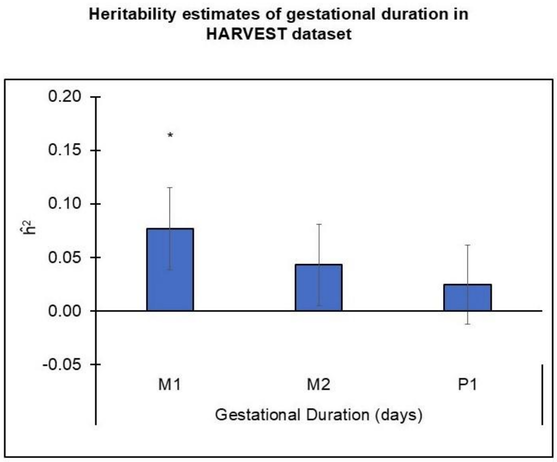

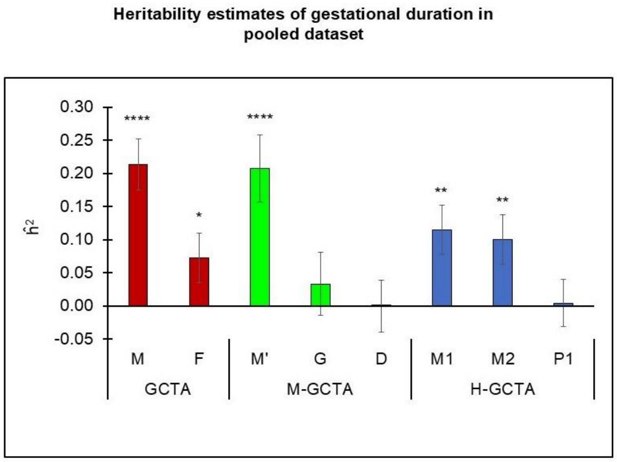

$\hat{h}^2$ estimation of fetal sex adjusted gestational duration in HARVEST dataset using our approach (H-GCTA). Estimated $\hat{h}^2$ of fetal sex adjusted gestational duration in pooled dataset is pasted for comparison (image on the right). Analysis was performed through GREML ($\alpha = -1.0$) using SNPs with MAF > 0.01.
