# Supplementary material for: Haplotype-based analysis distinguishes maternal-fetal genetic contribution to pregnancy-related outcomes
Source: PLoS Genet. 2025 Mar 10;21(3):e1011575. doi: 10.1371/journal.pgen.1011575 (PMC11918446; doi:10.1371/journal.pgen.1011575)
Supplement: Attachment — Submitted filename: Response_to_reviewers_comments.docx [file pgen.1011575.s037.docx]

**Responses to Reviewer's Comments**

**Comments to the Authors:
Please note here if the review is uploaded as an attachment.**

**Reviewer #1**: Srivastava and colleagues present H-GCTA, a new method for heritability analysis, that partitions SNP heritability based on contributions from maternal inherited, maternal not-inherited and paternal inherited alleles. They first demonstrate the method on simulated data, then applied to two real phenotypes (gestational duration and fetal size), using mother-child data from five cohorts.

In general, I have a positive view of the paper. I think the method is novel, the simulations are fair and extensive, and the real analysis will be on interest. I therefore have only minor comments. Please note that, having almost completed my review, I realised I had reviewed this paper before (back in 2020), so have added some extra brief comments at the bottom.

**Response:** We are thankful to the reviewer for reviewing the manuscript ‘twice’ and providing valuable comments. Previous and current comments substantially improved the quality of our manuscript.

\#####

Minor comments

1 - I notice you restrict estimates to be non-negative. Personally, I prefer to not restrict, because restricting introduces biases. However, I doubt it has a big impact here, and can help when you have correlated GRMs.

**Response:** Thank you for the suggestion. We agree that restricting the estimation could introduce bias. Variance components were estimated in unrestricted fashion for all simulated traits. We also compared the estimates without and with the constraint. We didn’t observe substantial difference except for the traits with correlated maternal-fetal effects. However, in the empirical data analyses, we encountered problems like “*non-positive definite var-covariance matrices*” and “*likelihood conversion*” particularly in the models with multiple GRMs including many with negligible estimates, for example, variance in birth length was primarily attributed to m1 whereas contribution of m2 or p1 were negligible. Therefore, we restricted estimation in our empirical data analyses. We have now added another set of analyses for pregnancy outcomes using pooled datasets with 5% MAF cut-off (Supplementary Table 26). In these analyses, we estimated variance components in an unrestricted manner.

2 - I wonder if necessary to use so many SNPs (i.e., imputed data). In general, using imputed is better, because it will capture more variation (and more causal variants, instead of variants tagging causal variants). However, doing so will lower precision relative to using just genotyped SNPs (both due to extra number of snps, and perhaps because imputed snps usually have more errors). Therefore, when sample size is not huge, you can find benefits using fewer snps (i.e., only directly genotyped, or even raising the MAF threshold to 0.05). However, this is just an idea, not a requirement.

**Response:** We agree with the reviewer on this point. However, our empirical data analyses were performed across different cohorts which used different arrays. Therefore, we needed to impute the data and then select a common set of SNPs across all cohorts. We believe that choosing different MAF cut-offs to check estimates and corresponding standard errors while analyzing empirical data partially explained the effect of number of markers on standard error.

3 - On a similar note, for your simulations, you could increase precision by considering a reduced genome (e.g., only chr 1-5). But as with 1, just an idea, not a requirement.

**Response:** Thank you for the suggestion. To better mimic the simulated traits with real ones, we expanded our simulations under various conditions using all polymorphic variants in a homogeneous population (ALSPAC) and a pooled dataset from five European cohorts. We also tested our simulations using only directly genotyped markers on a single chromosome, and the results were similar. However, we chose not to present these results to maintain simplicity and consistency between the simulated and empirical data analyses using whole-genome data.

4 - L180 - I would suggest rewording "Although, negative values of ĥ2 usually mean nothing and are considered as zero, they are important for interpretation of results for traits with negative correlation of maternal-fetal genetic effects." Maybe say "negative values of h2 usually correspond to noise". Also, I was a bit confused by this sentence, given that methods say you do restrict estimate to be non-negative.

**Response:** Thank you for the suggestion. We have modified the sentence accordingly. Also, estimates were only restricted in empirical data analyses not in the analyses of simulated data.

5 - I did not notice a software link, suggesting you used existing software. Therefore, I wondered how you created GRM from haplotype data? I think the easiest would be to save in bed format, where 0s and 1s are stored as 0 and 2 (doubling the values ensures LDAK / GCTA will standardize as per your formulae). It might be helpful for readers to explain how you performed the analyses. Please note that if you do not have bespoke software, and implementing is non-trivial, I am happy to add the feature to LDAK (i.e., construction of GRMs from haplotypes, outputed by Shapeit??)

**Response:** We sincerely thank the reviewer for offering the help to incorporate the feature in LDAK for construction of GRM from haplotypes. We have programs and scripts to split the haplotypes from phased vcf files, saving them into plink (0/2) format, creating and scaling the haplotype-based GRMs and running the estimation using GCTA and LDAK. We have now shared these programs and scripts through github (https://github.com/amitsrivastava-cchmc/H-GCTA/).

6 - I appreciate Figure 1. However, I wonder if the word "interface" gives impression you consider interactions between mother and father?

**Response:** Thanks for the appreciation. We understand that the word “interface” may appear a little confusing if considered beyond genetic aspects. Therefore, we have replaced the word “interface” to “shared genome” to make it simple.

7 - The simulation results figures. I am unsure whether you should highlight significance (wrt 0)? This is interesting for some comparisons (eg., where a method wrongly attributes variance). But in general, we are more interested in accuracy (closeness to true values).

**Response:** We agree with the idea that accuracy of estimates is more important, and we believe that point heritability estimates and associated standard errors provided that information with respect to their theoretically expected values. In our report, we present these p values to express the distinction of estimates against null, i.e. zero contribution.

8 - Figure 4 - Please ensure good resolution and clear labels (maybe the journal's conversion to pdf has damaged it). As p1 value is contact, can the x-axis simply be m1?

**Response:** We apologize for the low resolution of the figures (Reviewer 2 has the same concern); we improved the quality of the figures in this revised version. We also uploaded high-resolution figures as separate files to avoid problems during PDF conversion. In Figure 4, contribution of both m1 and p1change with varying levels of imprinting (POEs). With increasing level of imprinting of m1 contribution of m1 decreases whereas contribution of p1 increases to sum it up to true heritability i.e. 50%.

9 - The PC plot in Supp Figure 6 is a bit concerning, although partly because no reference genomes (e.g., you could overlay on 1000GP, so we can see the observed variation is small-scale, or global). However, while global variation might lead to variation, I believe it is unlikely to affect the relative values, which is the point of this study.

**Response:** Thank you for the suggestion. We have included the PCA plots anchored with 1000 genome samples (Supplementary Figure 6). Since 1000 genomes data in general lacks family/related samples, we anchored haplotypes created from the first allele of phased genotypes in phase3 1000 genomes data with m1 and p1 and haplotypes created from second allele of phased genotypes with m2. This analysis showed our study samples were relatively homogenous and overlapping closely with European reference samples.

10 - Supp Figure 7, would help to add your primary results (so can compare with those from replication data).

**Response:** Thank you for the suggestion. We have now pasted the main figures along with supplementary figure 7 for better comparison.

11 - Naturally, it would be interesting to see results from a non pregnancy trait (e.g. height) as comparison / a control.

**Response:** In the testing period of approach, we performed the analyses with human height and observed the estimates in agreement with other reports (M1 + M2 ≈ 55%) using 1% MAF cut-off in ALSPAC dataset.

Heritability estimates of Height in ALSPAC dataset

|  |  |
| --- | --- |
| M1 | 0.265313 |
| M2 | 0.29042 |
| P1 | 0.041083 |

\######

As noted, I eventually realised I previously reviewed this paper in 2020. I see that while generally positive, I criticised the use of only the "GCTA Model". Therefore, it greatly pleases me the authors listened to that criticism, and considered two alternative models. Thank you.

**Response:** We are extremely thankful for your comments previously and this time as well. These comments substantially improved the quality of our study.

\######

Signed Doug Speed

**Reviewer #2**: Srivastava et al. introduce an approach (H-GCTA) to partition the phenotypic variance of pregnancy outcomes into maternal transmitted, non-transmitted, and paternal transmitted alleles in mother-child pairs. Through simulation studies and real data analyses, the authors demonstrate that, compared to the M-GCTA method, the new H-GCTA can detect the contribution of parent-of-origin effects (POEs).

**Response:** We are thankful to the reviewer for taking the time to review our manuscript and providing valuable suggestions. These comments have not only substantially improved the quality of our manuscript but also helped us understand how our newly developed approach behaves in more complex and realistic scenarios.

My major comment concerns the significance of the study and the simulation design.
- The authors did not provide a publicly available tool for the approach, which limits its potential use.

**Response:** Thank you for this important feedback. We used only publicly available tools like GCTA and LDAK along with Bash and R scripts to run all the analyses. We have now shared our programs and scripts through github (https://github.com/amitsrivastava-cchmc/H-GCTA) which can be used for haplotype-based SNP-heritability estimation. Instructions for usage are provided in the README.md file.

- Regarding the simulation study:
1) Why was the contribution of POEs studied only for traits only with fetal effects? Can we simulate data with different levels of POEs also for traits with varying correlations between maternal and fetal genetic effects? This would help in understanding more complex traits that involve both correlated maternal and fetal genetic effects and POEs.

**Response:** Thank you for this suggestion. We have now added a new set of simulations for traits with correlated maternal-fetal genetic effects and parent-of-origin effects. We have simulated traits with average correlation of maternal and fetal effects = 1.0 with varying levels of POEs (0 ≤ m1/p1 ≤ 0.75). For simplicity, we assumed that all variants exhibited POEs. While GCTA and M-GCTA approach were unable to detect the contribution of POEs, H-GCTA clearly identified that variance attributable to m1 decreased with increasing levels of POEs. As expected, relative variance attributable to m1, m2 and p1 changes from $\hat{h}_{m1}^{2}:\hat{h}_{m2}^{2}:\hat{h}_{p1}^{2}=4:1:1$ in the absence of maternal imprinting to $\hat{h}_{m1}^{2}:\hat{h}_{m2}^{2}:\hat{h}_{p1}^{2}=1:1:1$ in case of complete maternal imprinting (please refer to main text, Supplementary Figure 5, and Supplementary Table 22).

2) It would be beneficial to calculate the true heritability attributable to maternal transmitted, non-transmitted, and paternal transmitted alleles in the simulated data (since we can compute the variance of each component relative to the variance of y), and then compare the results from GREML, LDAK-Thin, and LDAK-Weights to these true values.

**Response:** The expected values of the heritability attributable to maternal transmitted (m1), maternal non-transmitted (m2) and paternal transmitted (p1) haplotypes depend on the simulated conditions, and they can be calculated using the mathematical formulae provided in the supplementary figure 5. For example, assuming exclusive maternal or fetal traits with 50% heritability, GCTA will estimate approximately 12.5% contribution from fetal and maternal genome, respectively, due to shared genome between mother and fetus. Whereas M-GCTA should estimate 50% variance attributable to maternal genome and H-GCTA should estimate 25% variance attributable to m1 and m2 each for exclusive maternal traits. Likewise, M-GCTA should estimate 50% variance attributable to fetal genome and H-GCTA should estimate 25% variance attributable to m1 and p1 each for exclusive fetal traits. For traits with correlated maternal-fetal effects (rho = 1.0), GCTA should overestimate (50% variance attributable to both maternal and fetal genotypes) whereas M-GCTA should estimate equal contribution from M’, G and D (~17% attributable to each component) and H-GCTA should estimate h^2^ attributable to m1, m2 and p1 in a ratio of 4:1:1. Likewise, we can compare estimated values with all expected values in all simulated conditions. Although we didn’t provide a comprehensive comparison across all simulated conditions and approaches, we have discussed it in the text wherever needed.

3) Many observations from simulation studies can be derived mathematically. For example, given the assumed correlation between maternal and fetal genetic effects (\rho), one can explicitly derive how this correlation affects the contributions of m1, m2, and p1. As noted in line 203, when \rho = 1, the expected contributions of m1, m2, and p1 follow a 4:1:1 ratio; line 167 when \rho = 0, the expected contributions of m1, m2, and p1 follow a 2:1:1 ratio. These can be generalized to express the ratio as a function of (\rho). Similarly, given an assumed imprinting factor, one can derive how it affects the ratio.

**Response:** We agree with the reviewer that the expected values of the heritability attributable to maternal transmitted (m1), maternal non-transmitted (m2) and paternal transmitted (p1) can be derived mathematically. These mathematical relationships are summarized in Supplementary Figure 5. Below, we present the mathematical derivation of the expected contributions of m1, m2, and p1 as functions of (ρ) and an imprinting factor (γ). Because these derivations were built on a simplified model and may not capture the complexity of real data, we have opted not to include them in this article.

Considering $v$ variants with allele frequency $q$ following a distribution $\pi(q)$, and maternal ($a$) and fetal effect ($b$). Assuming $a\sim N[0,\alpha]$ and $b\sim N[0,\beta]$, and $\mathrm{cor}(a,b)=\rho$. With POE, we define imprinting factor $\gamma$, where the effect of maternal transmitted alleles ($b_{m}=b$) and paternal transmitted alleles ($b_{p}=\gamma b$). Therefore, the effect sizes of the m1, m2 and p1 alleles are:

| Alleles | $m1$ | $m2$ | $p1$ |
| --- | --- | --- | --- |
| Allelic effect | ${a+b}_{m}$ | $a$ | $b_{p}$ |

H1 (genetic variance attributable to m1):

$$h_{1}^{2}=\sum_{v} pq\left( a+b_{m} \right)^{2}=\sum_{v} pq\left( a+b \right)^{2}=\int_{q} pq \pi\left( q \right) \left( \alpha^{2}+\beta^{2}+2\alpha\beta\rho\right)dq$$

H2 (genetic variance attributable to m2):

$$h_{2}^{2}=\sum_{v} pq\left( a \right)^{2}=\int_{q} pq \pi\left( q \right) \left( \alpha^{2} \right)dq$$

H3 (genetic variance attributable to p1):

$$h_{3}^{2}=\sum_{v} pq\left( b_{p} \right)^{2}=\sum_{v} pq\left( \gamma b \right)^{2}=\int_{q} pq \pi\left( q \right)\left( \gamma\beta\right)^{2}dq$$

Minor comments:
- Line 152: The term "independent maternal-fetal genetic effects" is not accurate. A correlation = 0 does not imply independence.

**Response:** We agree that “a correlation = 0 does not imply independence.” There might be a confusion in our usage of independent maternal and fetal genetic effects which we defined as the independence of effect sizes of either same set or different sets of causal variants picked from a standard normal distribution in simulation. On the contrary, correlated maternal-fetal genetic effects were picked from a bivariate standard normal distribution with predefined correlation coefficient.

- Line 82: M-GCTA was introduced without citations.

**Response:** Thank you for the suggestion. We have incorporated the citations at appropriate places.

- In Figures 2, 3, 4, and 5, the y-axis labels are too densely packed, making it difficult to read the exact values.

**Response:** We have now modified the resolution of all figures including supplementary figures. We have also uploaded high-resolution figures (included in main text) as separate files to prevent any issues during PDF conversion.

**Have all data underlying the figures and results presented in the manuscript been provided?**
Large-scale datasets should be made available via a public repository as described in the *PLOS Genetics* [data availability policy](https://nam02.safelinks.protection.outlook.com/?url=http%3A%2F%2Fc05y1x9s.r.us-east-2.awstrack.me%2FL0%2Fhttp%3A%252F%252Fjournals.plos.org%252Fplosgenetics%252Fs%252Fdata-availability%2F2%2F010f0191fc2eed72-2f4894c7-bbdb-4b31-be42-2cc175cb0aac-000000%2F9oN_8hZHq-9vJrLGLa42vKpaF-E%3D176&data=05%7C02%7CAmit.Srivastava%40cchmc.org%7Cff3cedfc4b934da9e7e408dcd681fc85%7C680254d4278b4aa1aca8c2600f79c533%7C0%7C0%7C638621101005606100%7CUnknown%7CTWFpbGZsb3d8eyJWIjoiMC4wLjAwMDAiLCJQIjoiV2luMzIiLCJBTiI6Ik1haWwiLCJXVCI6Mn0%3D%7C0%7C%7C%7C&sdata=KRW53%2FvmRwFyOTBWh1FrNOJJnYBI7%2FsznrQEr%2BDQHi0%3D&reserved=0), and numerical data that underlies graphs or summary statistics should be provided in spreadsheet form as supporting information.

Reviewer #1: Yes

Reviewer #2: Yes

PLOS authors have the option to publish the peer review history of their article ([what does this mean?](https://nam02.safelinks.protection.outlook.com/?url=https%3A%2F%2Fc05y1x9s.r.us-east-2.awstrack.me%2FL0%2Fhttps%3A%252F%252Fjournals.plos.org%252Fplosgenetics%252Fs%252Feditorial-and-peer-review-process%2523loc-peer-review-history%2F1%2F010f0191fc2eed72-2f4894c7-bbdb-4b31-be42-2cc175cb0aac-000000%2FhJvqFs2YY2MxHr6LTMpHLSOSLUM%3D176&data=05%7C02%7CAmit.Srivastava%40cchmc.org%7Cff3cedfc4b934da9e7e408dcd681fc85%7C680254d4278b4aa1aca8c2600f79c533%7C0%7C0%7C638621101005615561%7CUnknown%7CTWFpbGZsb3d8eyJWIjoiMC4wLjAwMDAiLCJQIjoiV2luMzIiLCJBTiI6Ik1haWwiLCJXVCI6Mn0%3D%7C0%7C%7C%7C&sdata=e0kVQUbNb4ftmC7YaCADg1JLqTBFHMiYarm1cmJBITQ%3D&reserved=0)). If published, this will include your full peer review and any attached files.


If you choose “no”, your identity will remain anonymous but your review may still be made public.


**Do you want your identity to be public for this peer review?** For information about this choice, including consent withdrawal, please see our [Privacy Policy](https://nam02.safelinks.protection.outlook.com/?url=https%3A%2F%2Fc05y1x9s.r.us-east-2.awstrack.me%2FL0%2Fhttps%3A%252F%252Fwww.plos.org%252Fprivacy-policy%2F1%2F010f0191fc2eed72-2f4894c7-bbdb-4b31-be42-2cc175cb0aac-000000%2FLwSNRvdREBo7EI4F49O1icc1OcU%3D176&data=05%7C02%7CAmit.Srivastava%40cchmc.org%7Cff3cedfc4b934da9e7e408dcd681fc85%7C680254d4278b4aa1aca8c2600f79c533%7C0%7C0%7C638621101005623503%7CUnknown%7CTWFpbGZsb3d8eyJWIjoiMC4wLjAwMDAiLCJQIjoiV2luMzIiLCJBTiI6Ik1haWwiLCJXVCI6Mn0%3D%7C0%7C%7C%7C&sdata=vtrskesurk5bbgvtq1nAT3OvICw9OgAGqYQQE8lrO%2Bk%3D&reserved=0).

Reviewer #1: **Yes:** Doug Speed

Reviewer #2: No
